# Increased Expression of a MicroRNA Correlates with Anthelmintic Resistance in Parasitic Nematodes

**DOI:** 10.3389/fcimb.2017.00452

**Published:** 2017-11-06

**Authors:** Victoria Gillan, Kirsty Maitland, Roz Laing, Henry Gu, Neil D. Marks, Alan D. Winter, David Bartley, Alison Morrison, Philip J. Skuce, Andrew M. Rezansoff, John S. Gilleard, Axel Martinelli, Collette Britton, Eileen Devaney

**Affiliations:** ^1^Institute of Biodiversity, Animal Health and Comparative Medicine, College of Medical, Veterinary and Life Sciences, University of Glasgow, Glasgow, United Kingdom; ^2^Moredun Research Institute, Pentlands Science Park, Edinburgh, United Kingdom; ^3^Department of Comparative Biology and Experimental Medicine, Faculty of Veterinary Medicine, University of Calgary, Calgary, AB, Canada; ^4^Wellcome Trust Sanger Institute, Cambridge, United Kingdom

**Keywords:** parasitic nematode, *Haemonchus*, drug resistance, microRNA, ivermectin

## Abstract

Resistance to anthelmintic drugs is a major problem in the global fight against parasitic nematodes infecting humans and animals. While previous studies have identified mutations in drug target genes in resistant parasites, changes in the expression levels of both targets and transporters have also been reported. The mechanisms underlying these changes in gene expression are unresolved. Here, we take a novel approach to this problem by investigating the role of small regulatory RNAs in drug resistant strains of the important parasite *Haemonchus contortus*. microRNAs (miRNAs) are small (22 nt) non-coding RNAs that regulate gene expression by binding predominantly to the 3′ UTR of mRNAs. Changes in miRNA expression have been implicated in drug resistance in a variety of tumor cells. In this study, we focused on two geographically distinct ivermectin resistant strains of *H. contortus* and two lines generated by multiple rounds of backcrossing between susceptible and resistant parents, with ivermectin selection. All four resistant strains showed significantly increased expression of a single miRNA, *hco-miR-9551*, compared to the susceptible strain. This same miRNA is also upregulated in a multi-drug-resistant strain of the related nematode *Teladorsagia circumcincta*. *hco-miR-9551* is enriched in female worms, is likely to be located on the X chromosome and is restricted to clade V parasitic nematodes. Genes containing predicted binding sites for *hco-miR-9551* were identified computationally and refined based on differential expression in a transcriptomic dataset prepared from the same drug resistant and susceptible strains. This analysis identified three putative target mRNAs, one of which, a CHAC domain containing protein, is located in a region of the *H. contortus* genome introgressed from the resistant parent. *hco-miR-9551* was shown to interact with the 3′ UTR of this gene by dual luciferase assay. This study is the first to suggest a role for miRNAs and the genes they regulate in drug resistant parasitic nematodes. *miR-9551* also has potential as a biomarker of resistance in different nematode species.

## Introduction

Just as antimicrobial resistance poses a serious threat to the treatment of bacterial infections, anthelmintic resistance is a major challenge for the control of parasitic nematodes. This is particularly so in the livestock industry, with nematodes of small ruminants showing resistance to multiple anthelmintics (Kaplan and Vidyashankar, 2012). Three classes of drug are in common use in livestock species; the benzimidazole compounds, the cholinergic compounds (e.g., levamisole) and the macrocyclic lactones [e.g., ivermectin (IVM)]. In some parts of the world, including the United Kingdom, parasites resistant to all three drug classes have been identified (Sargison et al., 2007), posing a threat to the viability of livestock farming and compromising global food security. Of these broad-spectrum compounds, the macrocyclic lactones are the most recent and the most widely used. IVM was introduced to the animal health market in 1981 and within seven years, the first reports of resistance were recorded from South Africa (van Wyk and Malan, 1988). Since then, many studies have sought to determine the molecular mechanisms of IVM resistance. In the model nematode *Caenorhabditis elegans*, IVM targets the glutamate-gated chloride channels (GluCls) (Cully et al., 1994) causing paralysis of both the pharyngeal and body wall muscle in sensitive worms (Pemberton et al., 2001). High-level resistance to IVM in *C. elegans* is multigenic (Dent et al., 2000), requiring mutations in three separate genes encoding sub-units of the GluCls (*avr-14, avr-15*, and *glc-1*). While other genes have been implicated in IVM resistance in *C. elegans*, most of these modulate the resistance phenotype and are unable on their own to confer high-level resistance. Whether or not similar mechanisms underlie IVM resistance in parasitic nematodes is the focus of much debate (Gilleard and Beech, 2007).

Recent studies on the important livestock parasite *Haemonchus contortus* have implicated single nucleotide polymorphisms (SNPs) and other mutations in orthologs of genes known to be involved in IVM resistance in *C. elegans* (McCavera et al., 2009), but there is no consistent clear-cut association between the presence of particular SNPs and IVM resistance in different isolates. The situation is further complicated by known differences in the targets of IVM in *C. elegans* and *H. contortus*. For example, *glc-1* is not present in the *H. contortus* genome, while the parasitic species expresses two additional GluCl sub-units, *glc-*5 and *glc-6*, that are absent from *C. elegans* (Laing et al., 2013). IVM is also employed in mass drug administration campaigns for control of filarial infections, such as *Onchocerca volvulus* in humans (Osei-Atweneboana et al., 2011) and *Dirofilaria immitis* in dogs. In both these parasites, resistance is a growing concern (Bourguinat et al., 2015; Wolstenholme et al., 2015; Doyle et al., 2017).

In addition to SNPs, differences have been identified in the levels of expression of GluCls in IVM resistant worms (El-Abdellati et al., 2011) and in various subunits of levamisole-sensitive acetylcholine receptors (Kopp et al., 2009), in addition to P-glycoproteins and ABC drug transporters (Dicker et al., 2011; Raza et al., 2016), suggesting that changes in the composition of drug-sensitive channels and drug efflux pathways may be involved in resistance. The mechanisms responsible for such changes in gene expression are largely unexplored, although deletions/polymorphisms in gene regulatory regions (such as the 3′ untranslated region, 3′ UTR) have been reported (Rao et al., 2009; Neveu et al., 2010). In this paper, we adopt a novel approach to understanding anthelmintic resistance by investigating the profile of miRNAs in drug resistant and susceptible isolates of *H. contortus*. miRNAs are a class of small non-coding RNAs with important roles in regulating gene expression (Filipowicz and Sonenberg, 2015). First identified in *C. elegans* (Lee et al., 1993), they are present in animals, plants and viruses with roles in many fundamental aspects of development, as well as in diseases such as cancer (Adams et al., 2014). miRNAs exert their function by binding to complementary sequences most often in the 3′ UTR of the target genes, in the context of the RNA Induced Silencing Complex (RISC). miRNAs have been intensively studied in tumor cells where their expression is frequently dysregulated. Consequently they have been proposed as targets for novel chemotherapy as well as diagnostic biomarkers (Suzuki et al., 2015). miRNAs have also been implicated in drug resistance in a variety of tumor models, where they can act by modulating the abundance of drug metabolizing enzymes or drug transporters, permitting a more efficient efflux of drug in resistant cells, or by modulating various signaling pathways (To, 2013).

We recently described the miRNAs of *H. contortus* adult and infective L3 stages using a deep sequencing and bioinformatic approach, resulting in the identification of 192 miRNAs (Winter et al., 2012). Here, we build upon these observations to investigate miRNA expression in strains of *H. contortus* that are resistant or susceptible to IVM. These included the drug susceptible MHco3(ISE) strain and two IVM-resistant strains of different geographical origin, MHco4(WRS) from South Africa and MHco10(CAVR) from Australia. Additionally, we had access to a unique resource, two hybrid lines resulting from a series of backcrosses between the IVM susceptible parent and each of the IVM-resistant strains (Redman et al., 2012). These backcrossed worms display a genotype more similar to the susceptible parent, while exhibiting IVM resistance, indicative of introgression of resistance alleles into the susceptible background. We show that the level of a single miRNA is significantly up-regulated in parental and backcrossed resistant strains compared to the susceptible MHco3(ISE) and identify possible target mRNAs of this miRNA, the expression levels of which correlate inversely with miRNA levels. Moreover, the orthologous miRNA is present at increased levels in a multidrug resistant strain of the related nematode *Teladorsagia circumcincta* (MTci5) relative to an anthelmintic susceptible strain (MTci2). Our results suggest a novel paradigm to help explain the development of IVM resistance in parasitic nematodes.

## Materials and methods

### Ethics statement

Experimental infections were performed at the Moredun Research Institute, UK as described previously (Laing et al., 2013). All experimental procedures were examined and approved by the Moredun Research Institute Experiments and Ethics Committee (MRI E46 11) and were conducted under approved UK Home Office license (PPL 60/03899) in accordance with the 1986 Animals (Scientific Procedures) Act.

### Worm strains and backcrosses

The MHco3(ISE) strain is susceptible to all broad-spectrum anthelmintics (Roos et al., 2004) and was inbred to produce the material for the *H. contortus* genome sequencing project at the Wellcome Trust Sanger Institute (Laing et al., 2013). MHco4(WRS) originates from South Africa and is resistant to IVM and benzimidazole anthelmintics (van Wyk and Malan, 1988) and is also known as the White River Strain, while MHco10(CAVR) is an Australian strain otherwise known as the Chiswick AVermectin Resistant strain (Le Jambre et al., 1995) which is highly resistant to IVM but susceptible to benzimidazoles and levamisole. All strains were maintained at the Moredun Research Institute. Introgression of IVM resistance-conferring loci from both resistant strains into the genetic background of the susceptible strain MHco3(ISE) was carried out as described previously by Redman et al. (2012). In short, progeny from the crosses of the resistant strains with MHco3(ISE) underwent four rounds of back-crossing to MHco3(ISE), with IVM selection, to produce a fourth backcross generation: MHco3/4.BC_4_ [MHco3(ISE) crossed with MHco4(WRS)] and MHco3/10.BC_4_ [MHco3(ISE) crossed with MHco10(CAVR)]. In this manuscript the backcrossed strains are referred to as MHco3/4.BC_4_ and MHco3/10.BC_4_. For *T. circumcincta*, two strains were compared: a susceptible strain (MTci2) originally from Weybridge, that was isolated prior to the use of broad spectrum anthelmintics and a multi-drug resistant strain, MTci5, that shows decreased susceptibility to fenbendazole, levamisole, and IVM (Dicker et al., 2011). Female worms of these strains were available as frozen pellets from MRI.

### Re-establishment of MHco3/4.BC_4_ and MHco3/10.BC_4_

For this study, MHco3/4.BC_4_ and MHco3/10.BC_4_ were used after an additional three rounds of selection with IVM (Oramec™) at 0.2 mg/Kg, with one exception. To re-establish MHco3/4.BC_4_ and MHco3/10.BC_4_, sheep were infected with 5,000 L3 of the appropriate line and treated with IVM at either 0.1 or 0.2 mg/Kg. At the first passage, no L3 were obtained from fecal cultures from the sheep infected with MHco3/10 BC_4_ treated at 0.2 mg/Kg IVM. Consequently, for the next round of selection, the input L3 came from the animal treated with IVM at 0.1 mg/Kg. The origin and maintenance of MHco3/4.BC_4_ and MHco3/10.BC_4_ is described in data shown in Table S1.

### Preparation of samples for microarray analysis

Three sheep were infected *per os* with 5,000 L3 of each of the strains, MHco3(ISE), MHco4(WRS), MHco10(CAVR), MHco3/4.BC_4_, and MHco3/10.BC_4_ and were not drug treated. An additional three sheep per strain were infected with only the IVM-resistant strains MHco4(WRS), MHco10(CAVR), MHco3/4.BC_4_, and MHco3/10.BC_4_ and treated with IVM at 0.2 mg/Kg 7 days prior to euthanasia. Infected sheep were euthanized at 28 days post-infection. Surviving worms were isolated from the abomasum, washed extensively in PBS and picked into groups of 20 males or 20 female worms and immediately snap frozen in liquid nitrogen. RNA was prepared from each sample individually by grinding the pellet using a liquid nitrogen-cooled pestle and mortar and standard Trizol® protocol (Invitrogen). RNA quality and quantity was assessed using a spectrophotometer and Bioanalyzer (Agilent 2100 Bioanalyzer). 7.5 μg of male RNA and 7.5 μg of female worm RNA from paired samples (i.e., from the same sheep) was pooled and sent on dry ice to LC Sciences (Houston, Texas) for microarray. The arrays contained 609 predicted miRNA sequences from *H. contortus* (Winter et al., 2012), 369 sequences from *C. elegans* miRNAs (miRBase 16) and 50 control sequences. The array was probed with triplicate biological samples of pooled male and female RNA labeled with Cy5. miRNA expression was analyzed in different worm groups using two methods, ANOVA (Analysis of Variance) and *t*-test, and the degree of significance evaluated by comparing between-group variation with within-group variation. For an experiment involving more than two sample groups, ANOVA was first applied to produce a miRNA expression profile overview across all samples, then *t*-tests were performed to identify significantly differentiated miRNAs among all combinations of two groups.

### Life cycle microarray

To ascertain the expression profile of *hco-miR-9551* throughout the *H. contortus* life cycle, data from a different microarray experiment were analyzed. This array contained exactly the same sequences as described above but the samples used to probe the array were all isolated from MHco3(ISE) worms, as follows: adult male, adult female, L4 isolated at day 7 post-infection, L3, *in vitro* activated L3 (exsheathed and cultured for 24 h in RPMI-1640 at 37°C and 5% CO_2_ in air) and gut-enriched tissue (dissected from adult female worms). Biological triplicates of RNA were prepared for each sample as described previously, but in this experiment RNA was labeled with Cy3.

The microarray dataset supporting the expression of miRNAs in resistant vs. susceptible *H. contortus* can be found on NCBI GEO (https://www.ncbi.nlm.nih.gov/geo/): Series GSE79542. The microarray dataset showing the expression of miRNAs across the *H. contorus* life cycle can be found at (https://www.ncbi.nlm.nih.gov/geo/): Series GSE101501.

### qRT-PCR

For qRT-PCR analyses, 1 μg of DNase I®-treated (Ambion) total RNA from female, male worms or L3 was used in polyadenylation and reverse transcription reactions (plus and minus enzyme) using the miRNA 1stStrand cDNA Synthesis® protocol (Agilent Technologies). Single RNA samples were also processed for qRT-PCR from female MHco3(ISE) worm gut tissue, from *in vitro* released eggs/L1 of MHco3 (ISE) worms or from parasite excretory-secretory (ES) products from MHco3/4.BC_4_ and MHco3/10.BC_4_ worms. qPCR was carried out following the miRNA qPCR Master Mix® protocol (Agilent Technologies) and results analyzed using MxPro qPCR Software, Version 4.10. Results were normalized to *hco-miR-50*, which was found by microarray to be present at similar levels throughout all the experimental conditions examined. The sequences of the HPLC-purified oligonucleotide primers (Eurofins) are as follows: *hco-miR-50*–5′-TGATATGTCTGGTATTCTTGG-3′; *hco-miR-9551*–5′-CACAGCATTTTACTGAGCC-3′. Agilent Universal Reverse miRNA primer® was used in all reactions.

Unless stated otherwise, qRT-PCR was performed on three biological samples and each PCR was carried out in triplicate. Lin-Reg® software (Ramakers et al., 2003; Ruijter et al., 2009) was used to calculate PCR efficiency using linear regression. These data were then processed by REST2009® software (qiagen.com) (Pfaffl, 2001; Pfaffl et al., 2002), which uses a mathematic model to account for any differences in PCR efficiencies between normaliser genes and genes of interest (http://rest.gene-quantification.info). This allows compensation for variations in starting material thus providing more accurate quantification of gene expression.

### Bioinformatic prediction of mRNA targets and pathway analysis

For this analysis two *in silico* 3′ UTR libraries were combined to generate a single searchable database. The first library contained 3′ UTR sequences predicted from the *H. contortus* transcriptomic data (Laing et al., 2013). This library contained 14,507 predicted 3′ UTRs from 11,264 genes and thus represented only ~52% of the genome (*n* = 21,799). The predicted 3′ UTRs ranged in length from 1–15,355 nt, although the majority were between 100 and 1,100 nt. 3′ UTRs of 50–1,000 nt were selected (11,328 sequences representing 8,992 genes) and combined with data from a second library that contained 1 kb of downstream sequence from each predicted gene in the genome, or sequence up to the end of a scaffold. As it was based on transcriptomic data, the first 3′ UTR database was considered the more reliable and sequences from the downstream database were used to “fill in” the missing 3′ UTRs.

The combined library, representing 3′ UTRs of 93% of all predicted *H. contortus* genes, was screened using the following online miRNA target prediction algorithms: PITA (Kertesz et al., 2007) (with default settings: seed sequence of 8 bases, ddG<−10^3^), miRANDA (Enright et al., 2003) (filtered for scores >145 and energies <−10), and RNAhybrid (Rehmsmeier et al., 2004) (*p* < 0.1 and energies <−22). Only 3′ UTRs that scored positive with all three programmes were retained. These were further analyzed to ensure that the binding sites predicted by each programme overlapped, resulting in a set of “high confidence” target mRNAs. These transcripts were then assessed using previously created RNAseq datasets generated from triplicate biological replicates of 20 pooled females worms of the MHco3(ISE), MHco4(WRS), and MHco10(CAVR) strains. Reads were mapped to the most current version (version 3) of the MHco3 *H. contortus* genome assembly (Laing et al., 2013) using Tophat2 (Kim et al., 2013). The following command line parameters were used: *-I 40000 -r 200 -p 6 -a 6 -g 1 -N 5 –read-gap-length 3 –read-edit-dist 8 –no-discordant –no-mixed –min-intron 10 –microexon-search –mate-std-dev 40 –library-type fr-unstranded*. A higher allowance for polymorphism between mapped RNAseq reads and the MHco3 genome assembly was allowed to ensure reads of other strains were not discarded due to the numbers of polymorphic sites. Mapped reads were counted using the *htseq-count* tool from HTSeq (Anders and Huber, 2010). DESeq2 was then used to call differential expression between strains of interest using default settings (Love et al., 2014). Transcripts that were down-regulated in both or one resistant strain compared to the susceptible MHco3(ISE) parental strain were identified. Transcriptomic data can be accessed via https://data.mendeley.com/datasets/mwvsjgt9jd/draft?a=2e2cfcc5-589f-4f93-ab6c-9330f92d13e8 (Rezansoff and Gilleard, unpublished).

Additionally, the top 400 predicted targets of *hco-miR-9551* derived from PITA, miRANDA, and RNAhybrid were used in pathway analysis using DAVID bioinformatics resources (Huang da et al., 2009). *C. elegans* orthologs of the predicted *H. contortus* genes were identified and used to assemble a gene list which was entered into the online DAVID tool (https://david.ncifcrf.gov/) where this list was converted into associated biological pathways based on gene-annotation enrichment analysis. *p* values were adjusted for multiple testing to control the false discovery rate, using the Benjamini-Hochberg procedure (Anders and Huber, 2010) and pathways with a score of *p* < 0.05 were considered significant.

### miRNA/mRNA interactions in HEK293 cells

3′ UTRs from predicted *hco-miR-9551* target genes HCOI00821400, HCOI00084600, and HCOI01910900 were amplified from *H. contortus* genomic DNA, isolated as described previously (Winter et al., 2013), using PfuUltra II Fusion HS DNA Polymerase (Agilent) and the following primer sequences (restriction sites underlined);

HCOI00821400 F *Not I* 5′GCGGCCGCCGTTGTTCCCATGCCACTTG-3′

HCOI00821400 R *Not I* 5′-GCGGCCGCGGATTCAAATCAGAAGC-3′

HCOI00084600 F *Not I* 5′-GCGGCCGCGCCCTACAATGTCTTCGCC-3′

HCOI00084600 R *Not I* 5′-GCGGCCGCGTGAGCAGGAAACAGC-3′

HCOI01910900 F *Not I* 5′-GCGGCCGCAGATTCAACATCCTCT-3′

HCOI01910900 R *Not I* 5′-GCGGCCGCTGATACTGGCTTTCCTCCA-3′

Products were cloned into pCR2.1-TOPO (Invitrogen) and then subcloned into the *Not* I site downstream of firefly luciferase in pMir-Target (Origene). Similarly, a 298 bp section of the *hco-miR-9551* locus was amplified from *H. contortous* genomic DNA using the following primer sequences, mir-9551 F *Kpn I* 5′-GGTACCGTTACTTGCCGAT-3′ and mir-9551 R *Kpn I* 5′-GGTACCTGTCTGTCTCATCA-3′. This product was cloned into pCR2.1-TOPO and subcloned into vector pEGFP-N1 (Clontech) using *Kpn* I to generate plasmids in the forward and reverse orientation.

HEK293 cells were maintained as described previously (Winter et al., 2015). For transfections, 1 × 10^4^ cells/ml were seeded into the wells of 96-well plates in a volume of 100 μl using Dulbecco's Modified Eagle's Medium (containing 4500 mg/L glucose and sodium bicarbonate, Sigma D5671). Cells were transfected after 24 h when they were ~50% confluent using Lipofectamine LTX (Invitrogen) with 50 ng of a *mir-9551-*containing plasmid in either the forward or reverse direction, 6.25 ng of the relevant pMir-Target-derived plasmid, and 0.5 ng of phRG-TK (Renilla luciferase, Promega). Transfections were performed following the manufacturer's protocol for HEK293 cells. DNA was diluted to a volume of 20 μL using Opti-MEM I Reduced Serum Medium (Invitrogen) and 0.35 μL Lipofectamine LTX Reagent added. After incubation at room temperature for 30 min 20 μL of the DNA-Lipofectamine complex was then added directly to the cells in each well. Cells were grown for 48 h then analyzed using a Dual Luciferase Assay kit (Promega) following the manufacturer's protocol with six replicates used per test condition.

## Results

### A single miRNA is differentially expressed in all IVM-resistant strains of *H. contortus* analyzed

A microarray consisting of all identified *H. contortus* miRNAs (Winter et al., 2012) and all *C. elegans* miRNAs (miRBase 15) was constructed (LC Sciences). The array was probed with a 1:1 mix of RNA from male and female *H. contortus* worms recovered from three individual sheep per strain, yielding triplicate biological replicates of IVM sensitive strain MHco3(ISE) and IVM resistant strains MHco4(WRS) and MHco10(CAVR) and the relevant backcrosses, MHco3/4.BC_4_ and MHco3/10.BC_4_ (see section Materials and Methods for details and nomenclature). The backcrossed lines have been described previously (Redman et al., 2012) and had undergone three additional rounds of selection with IVM when used in the current study. For the four IVM-resistant strains, worms were also collected from three sheep per strain that had been treated with 0.2 mg/kg of IVM 7 days prior to worm collection.

**Figure 1:**
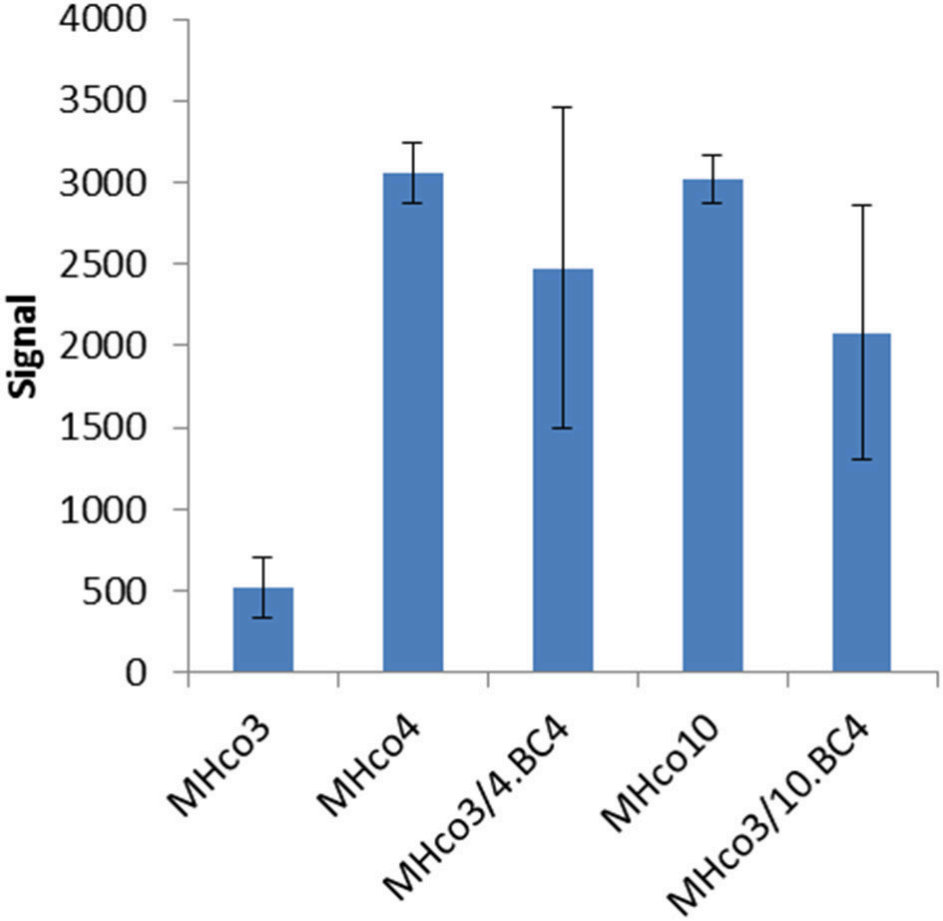
Microarray signal for *hco-miR-9551* from triplicate biological replicates of worms from five different strains. Equal amounts of RNA were pooled from 20 male and 20 female *H. contortus* isolated from three individual animals per strain and were labeled with Cy5 and used to probe the array. Results are expressed as mean values ± *SD*.

**Figure 2:**
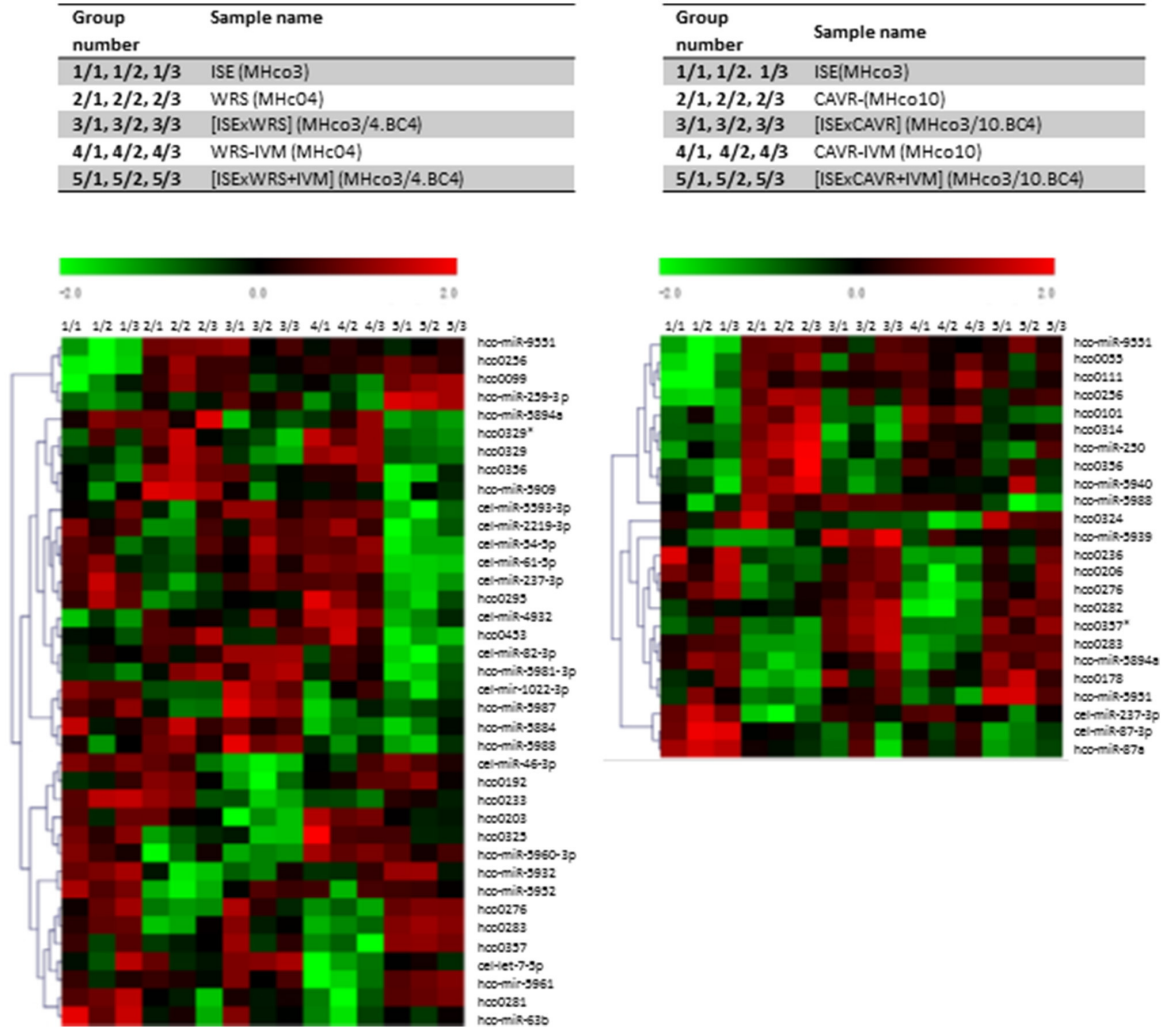
Microarray Heat Map. Heat maps were generated to depict miRNA expression between groups. Green, under expression; red, overexpression. Data shown depicts miRNAs showing significantly different levels of expression (*p* ≤ 0.01) between resistant and susceptible strains of *H. contortus* using ANOVA which was applied to produce a miRNA expression profile overview across all samples. ^*^Indicates star strand of miRNAs not in miRBase.

Analysis of the microarray data demonstrated similar expression levels of most miRNAs between IVM resistant and sensitive strains. However, a single miRNA, *hco-miR-9551*, was found at ~6-fold greater abundance in the IVM-resistant parental strains MHco4(WRS) and MHco10(CAVR) compared to the susceptible parent. In the backcrossed lines, MHco3/4.BC_4_ and MHco3/10.BC_4_, *hco-miR-9551* was also up-regulated 4- to 4.5-fold compared to IVM susceptible MHco3(ISE) worms (*p* < 0.05 for all samples; see Table 1, Figure 1). No other miRNA showed the same consistent up-regulation in every IVM-resistant strain and no miRNA was significantly decreased in all resistant strains (Figure 2). To investigate whether the expression of *hco-miR-9551* was further induced by exposure to IVM, the same microarray was probed with RNA isolated from IVM-resistant worms recovered from sheep that had been treated with IVM 7 days previously. No significant difference in *hco-miR-9551* expression levels was observed in any of the resistant strains compared to those recovered from untreated sheep (see Table 2 for details). Thus, *hco-miR-9551* does not appear to be further induced in response to IVM.

**Table 1:** Microarray signal for *hco-miR-9551* in *H. contortus* strains.

**Isolate**	***hco-miR-9551* signal ± *SD***	**Log_2_ fold increase in expression**	***P*-value**
MHco3(ISE)	517 ± 185		
MHco4(WRS)	*3, 054*±183	2.56	1.50 × 10^−2^
MHco3/4.BC_4_	*2, 475*±982	2.26	1.68 × 10^−2^
MHco10(CAVR)	*3, 016*±149	2.54	1.51 × 10^−2^
MHco3/10.BC_4_	*2, 081*±780	2.01	2.44 × 10^−2^

*Microarray signal represents mean ± SD of three biological replicates. p-value determined by t-test. Log_2_ fold increase for all strains is expressed relative to MHco3(ISE) susceptible strain*.

**Table 2:** Microarray signal for *hco-miR-9551* in all resistant strains plus/minus IVM treatment.

**Strain ± IVM**	**Mean microarray signal ± *SD***
MHco4 (WRS)	3,054 ± 183
MHco4 (WRS)+IVM	1,732 ± 256
MHco3/4.BC4	2,475 ± 982
MHco3/4.BC4 + IVM	1,636 ± 340
MHco10 (CAVR)	3,016 ± 149
MHco10 (CAVR)+IVM	2,035 ± 425
MHco3/10.BC4	2,081 ±780
MHco3/10.BC4 + IVM	2,456 ± 732

*Microarray signal represents mean ± SD of three biological replicates. p > 0.05 for all comparisons of ± IVM for any one strain*.

### qRT-PCR analysis confirms that *hco-miR-9551* expression is altered in IVM-resistant worms

**Figure 3:**
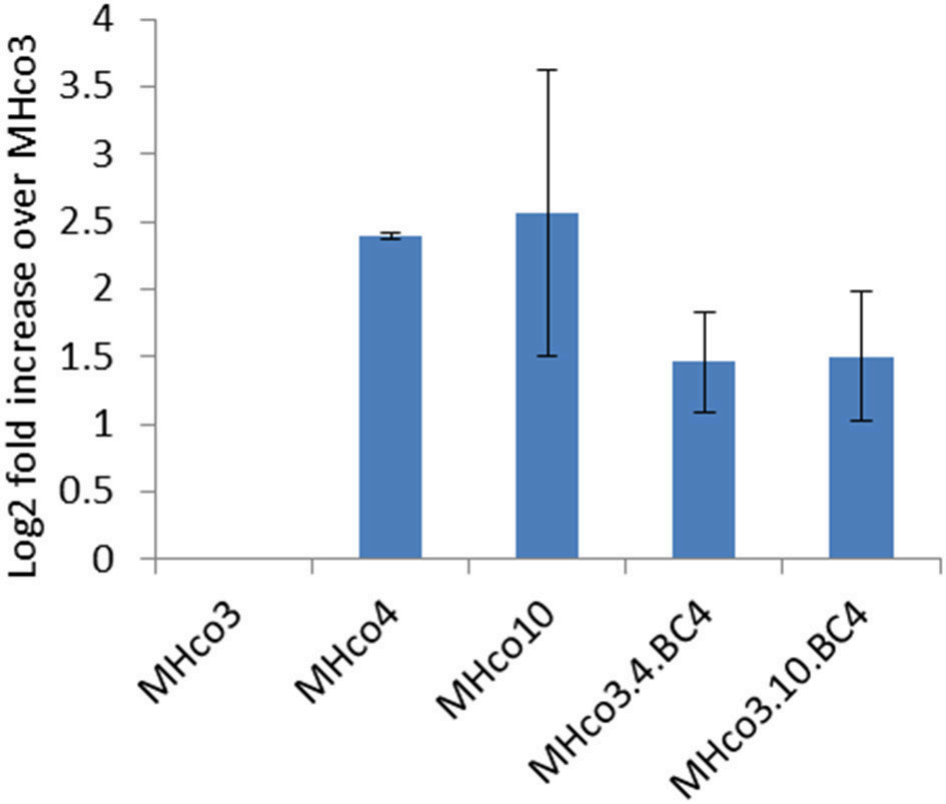
Expression of *hco-miR-9551* is upregulated in female worms of all four resistant strains. Graph shows the mean log_2_ fold increase + *SD* in *hco-miR-9551* levels in female worms of four IVM-resistant strains relative to the MHco3(ISE) susceptible strain, quantitated by RT-PCR. Triplicate biological samples of female worm RNA were analyzed for each strain. Data were normalized to expression levels of *hco-miR-50* and levels of *hco-miR-9551* expressed in resistant worms relative to that in MHco3(ISE). MxPro data were analyzed to ensure equal efficiency of reactions using Lin-Reg® followed by REST2009®. Results are presented using the corrected data.

**Figure 4:**
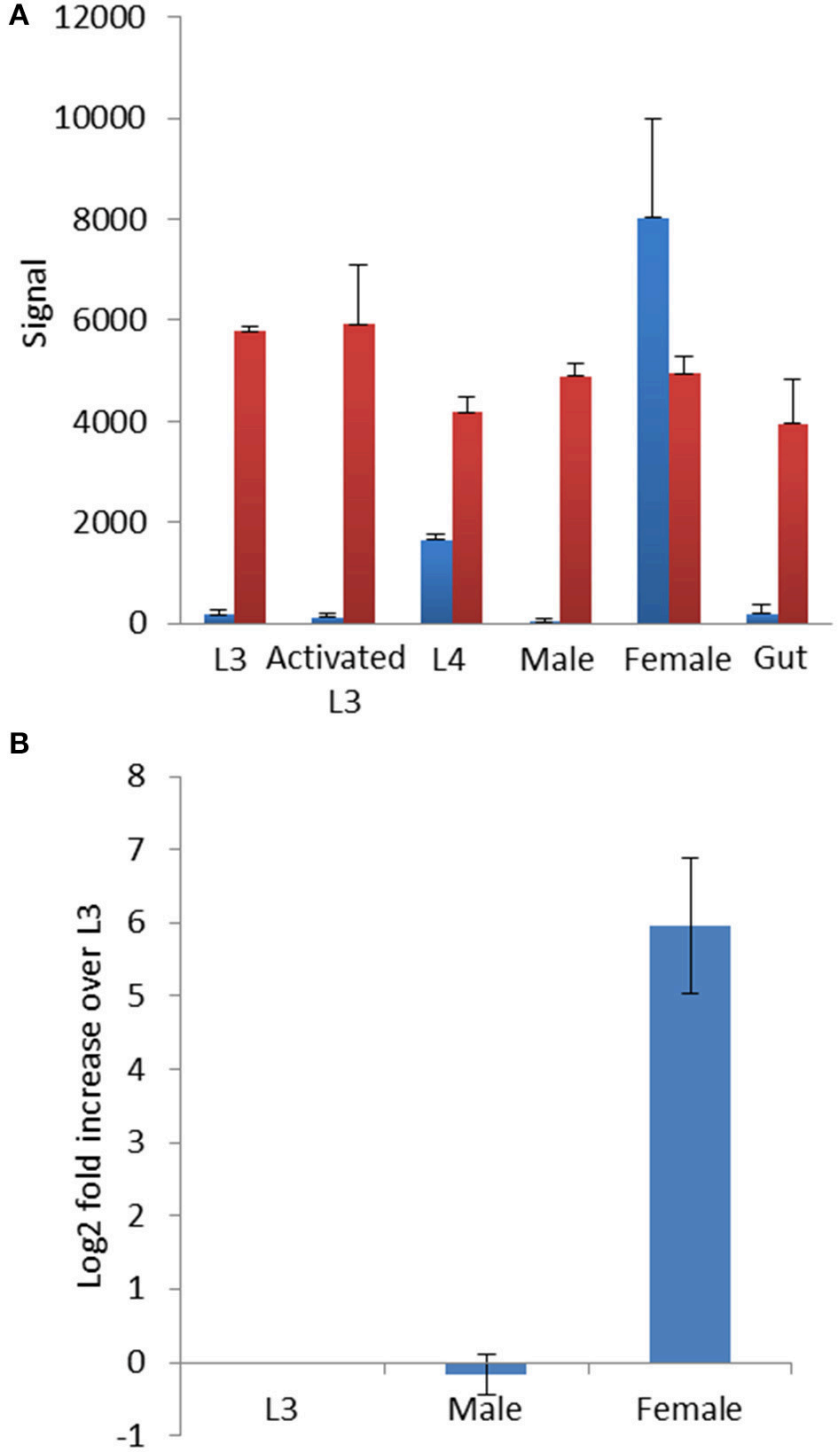
**(A)** *hco-miR-9551* is developmentally regulated in MHco3(ISE) worms. Signal from microarray probed with triplicate samples of different life cycle stages of MHco3(ISE) *H. contortus*. Graph shows the signal ± *SD* for *hco-miR-9551* in blue and a constitutively expressed miRNA, *hco-miR-50* in red. In this array, RNA was labeled with Cy3. **(B)** Expression profile of *hco-miR-9551* by qRT-PCR. qRT-PCR of MHco3(ISE) life cycle stages using triplicate biological replicates of L3, adult males or adult female worms. Graph shows the log_2_ fold increase over L3 normalized to *hco-miR-50*. MxPro data were analyzed to ensure equal efficiency of reactions using Lin-Reg® followed by REST2009®. Results are presented using the corrected data.

As the probes for the array consisted of a mixture of male and female adult worms, we investigated whether the expression of *hco-miR-9551* varied between sexes and life cycle stages. Additional RNA samples were prepared in triplicate from batches of male, female and L3 worms of all five strains and *hco-miR-9551* levels measured by qRT-PCR relative to *hco-miR-50*, a constitutively expressed miRNA (see microarray dataset). The results for female worms broadly confirmed those from the original microarray; MHco4(WRS) and MHco10(CAVR) worms showed a 5- to 6-fold increase over MHco3(ISE) worms, while the backcross lines MHco3/4.BC_4_ and MHco3/10.BC_4_ showed ~a 2- to 3-fold increase in expression of *hco-miR-9551* (Figure 3). In male worms and L3 larvae, levels of *hco-miR-9551* were not significantly different between susceptible and resistant strains examined by qRT-PCR (data not shown). The results for these life cycle stages were much less consistent, reflecting the low levels of *hco-miR-9551* in these stages (see Figure 4A). Thus, *hco-miR-9551* appears to be a miRNA that is significantly up-regulated in female worms of all IVM-resistant strains tested in this study.

### *hco-miR-9551* is sex-linked in MHco3(ISE) worms

As qRT-PCR analysis suggested that *hco-miR-9551* was highly expressed in female worms only, we investigated its expression in greater detail. Data were first analyzed from an identical microarray that had been probed with samples of RNA isolated from multiple life cycle stages of MHco3(ISE) worms. These included L3, *in vitro* activated L3 (exsheathed and cultured for 24 h, see section Materials and Methods for detail), L4 recovered at 7 days post-infection, male or female worms, or a preparation of gut tissue dissected from female worms (Marks et al. unpublished, but see microarray data set for details). This analysis confirmed that *hco-miR-9551* was expressed in female worms of the susceptible MHco3(ISE) strain; male worms, L3, *in vitro* activated L3 or gut tissue did not express significant levels of *hco-miR-9551* (see Figure 4A). The first signs of expression were evident in L4 stages recovered at 7 days post-infection, suggesting that this miRNA is developmentally regulated following infection of the mammalian host.

Expression levels of *hco-miR-9551* were confirmed by qRT-PCR in triplicate biological replicates of male, female and L3 MHco3(ISE) worms. The data in Figure 4B show *hco-miR-9551* levels relative to *hco-miR-50* and confirm that *hco-miR-9551* is essentially specific to female worms in the MHco3(ISE) strain with very low levels of expression in male worms or in L3. To determine if *hco-miR-9551* is expressed in eggs developing within adult female worms, further qRT-PCR studies were carried out. No detectable signal was observed for *hco-miR-9551* in a pool of eggs/L1 larvae collected from *in vitro* culture, nor was it detected in gut tissue isolated from single adult females, while a control miRNA could be amplified from all samples (data not shown). However, *hco-miR-9551* was identified in a small RNA library prepared from excretory-secretory (ES) products of adult MHco3(ISE) worms and could be amplified by PCR from ES products of cultured adult worms of both backcrosses (Gu et al. unpublished data), suggesting it may be released from the secretory system and/or female gonad.

### Characterisation of the *hco-miR-9551* locus

The *hco-miR-9551* locus is located on scaffold_496 (Laing et al., 2013) and see WormBase ParaSite (http://parasite.wormbase.org). This scaffold is just over 140 Kb in length and encodes eight protein-coding gene models. Five of these gene models have predicted orthologs in *C. elegans*, all of which reside on the X chromosome, suggesting scaffold_496 is derived from the *H. contortus* X chromosome. All eight gene models are encoded on the reverse strand, while the *hco-miR-9551* precursor sequence is encoded on the forward strand, lying 3,866 bp downstream (3′ end) of gene model HCOI01030600.t1 and 10,152 bp upstream (5′ end) of gene model HCOI01030500.t1 (See scaffold in Figure S1). Evidence in the literature has shown that that some miRNAs can affect the expression of genes in close proximity, providing a local negative feedback mechanism (van Rooij et al., 2009; Dill et al., 2012; Truscott et al., 2014; Yuva-Aydemir et al., 2015) and interestingly, both these genes are expressed at a lower level in MHco4(WRS) and MHco10(CAVR) than in MHco3(ISE) female worms, although the differences do not reach significance (data not shown). Searching for *hco-miR-9551* binding sites in both genes demonstrated that HCOI01030600.t1 contained four potential sites in the 3′ UTR and one within the open reading frame. The predicted products of these genes are of unknown function and they have no conserved domains or orthologs in *C. elegans*. They do, however, have orthologs in other clade V parasitic nematodes including *Oesophagostomum dentatum* and *Ancylostoma ceylanicum* (for HCOI01030600.t1) and *A. ceylanicum* and *Dictyocaulus viviparus* (for HCOI01030500.t1).

### *miR-9551* is specific to clade V parasitic nematodes

There are no orthologs of *hco-miR-9551* documented in miRBase, but scrutiny of the genomes of other clade V parasitic species identified an orthologous sequence in several species. In each case, RNA-fold analysis of the parasite *mir-9551* using Mfold (http://unafold.rna.albany.edu/?q$=$mfold/RNA-Folding-Form) (Zuker, 2003), supports a hairpin structure with folding energies consistent with miRNA structure (data shown in Table S2). No such sequence was found in free-living nematodes, such as *Caenorhabditis* species and examination of parasitic nematode sequences from species belonging to clade I, III or IV, failed to identify any orthologs of *hco-miR-9551*. Thus, *hco-miR-9551* appears to be specific to clade V parasitic nematodes belonging to the sub-order Strongylida. The only miRNA present in miRBase showing any degree of homology with *hco-miR-9551* is *miR-308-3p* from various species of *Drosophila*, which shares the same seed sequence (nucleotides 2-7) with *hco-miR-9551*.

### *miR-9551* is also upregulated in multi-drug resistant *T. circumcincta*

**Figure 5:**
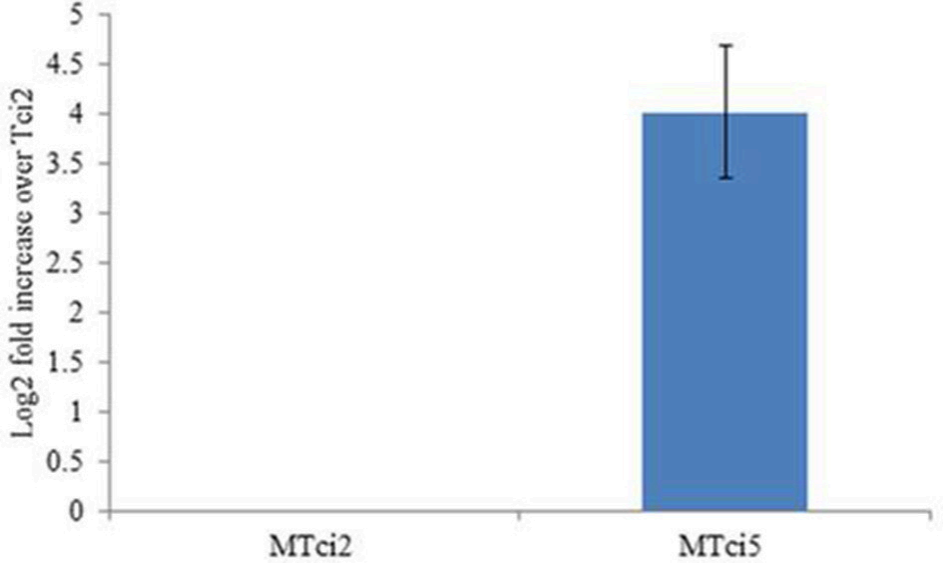
Expression of *miR-9551* is upregulated in resistant *T. circumcincta*. Triplicate biological samples of MTci2 and MTci5 adult female worms (susceptible and resistant, respectively) were analyzed by qRT-PCR for changes in the expression levels of *tci-miR-9551*. Graph shows the log_2_ fold increase of *tci-miR-9551* normalized to *tci-miR-50*. As above, efficiency of the reactions were analyzed using Lin-Reg® followed by REST2009® and results are presented using the corrected data.

To investigate whether upregulation of *miR-9551* occurred in other parasitic nematodes showing resistance to IVM, we focused on the related strongylid *T. circumcincta*. Strain MTci2 is susceptible to anthelmintic, while strain MTci5 shows resistance to IVM, fenbendazole and levamisole (Dicker et al., 2011). Expression levels of the *T. circumcincta hco-miR-9551* ortholog, *tci-miR-9551*, were compared in the drug sensitive strain (MTci2) compared to the multi-drug resistant strain (MTci5). As can be seen from Figure 5, the expression of *tci-miR-9551* was 16-fold higher in drug resistant *T. circumcinta* (*p* = 0.037).

### Identifying mRNA targets of *hco-miR-9551* using bioinformatic and comparative genomic approaches

Determining the function of a specific miRNA is difficult, even in organisms such as *C. elegans*, where genetic manipulation is feasible. In an attempt to define the biological roles of *hco-miR-9551*, we identified target mRNAs using computational programmes that search for miRNA binding sites. We screened an *in silico* 3′ UTR library using three separate programmes, miRANDA, PITA, and RNAhybrid to increase the likelihood of identifying true mRNA targets (see section Materials and Methods for details of settings). Only those 3′ UTRs scoring positive in all three programmes were considered further. The number of 3′ UTRs containing predicted *hco-miR-9551* binding sites varied markedly from 2,255 using MIRANDA to 653 and 435 with RNAhybrid and PITA, respectively. In all, 73 UTRs were predicted by all three programmes to have *hco-miR-9551* binding sites, of which 68 had overlapping binding sites predicted by all three programmes and therefore were considered “high confidence” targets (data shown in Table S3).

As miRNA expression frequently shows an inverse correlation with target mRNAs, we aimed to narrow down potential mRNA targets for further analysis, by scrutinizing a transcriptomic dataset prepared from adult female worms of the MHco3(ISE), MHco4(WRS) and MHco10(CAVR) strains (Rezsanoff, Laing and Gilleard, unpublished). Of the 26,064 genes in this transcriptomic dataset, a total of 694 were down-regulated in both MHc04(WRS) and MHc010(CAVR) female worms compared to MHc03(ISE). We focused on the expression profile of the 68 genes with predicted *hco-miR-9551* binding sites in both resistant strains, MHc04(WRS) and MHc010(CAVR), compared to the susceptible strain, MHco3(ISE). Of these 68 genes, three showed significantly reduced expression in both resistant strains (Benjamini-Hochberg adjusted score *p* < 0.05) as listed in Table 3A. The expression of several other mRNAs was reduced in only one of the resistant strains compared to the IVM-susceptible female worms (see Table 3B). Of the three genes down-regulated in both resistant strains, HCOI00084600 is a predicted RNA-binding protein in both *H. contortus* and *C. elegans*, HCOI00821400 contains a ChaC domain with predicted gamma-glutamyl cyclotransferase (GGCT) function, while HCOI01910900 encodes a hypothetical protein. Two genes were significantly down-regulated only in MHco4(WRS) worms; these encoded a predicted protein with a 7-transmembrane G-protein-coupled receptor domain (HCOI00499800), the *C. elegans* homolog of which (*ser-4*) shows similarity to mammalian serotonin receptors. The second gene is a probable vacuolar ATPase (HCOI01954200). In MHco10(CAVR) worms, a collagen gene (HCOI02065400), a probable histone acetyltransferase gene (HCOI00706800) and a protein with a predicted glycolipid-transfer domain (HCOI0277200) were all significantly down-regulated. The putative *C. elegans* homologs of these genes and their RNAi phenotypes are shown in Tables 4A and B.

**Table 3A:** Predicted mRNA targets of *hco-miR-955*1 down-regulated in both IVM-resistant strains, MHco4(WRS), and MHco10(CAVR) compared to the susceptible MHco3(ISE) strain.

**mRNA**	**HCOI00084600**	**HCOI01910900**	**HCOI00821400**
Predicted protein	RNA recognition motif	Hypothetical	ChaC domain
Normalized Expression MHco3(ISE)	243.64	9.39	37.51
Normalized Expression MHco4(WRS)	68.037	1.367	18.23
Log_2_ fold change	−1.84	−2.78	−1.04
Adjusted *P* value	0.001	0	0.044
Normalized Expression MHco10(CAVR)	47.625	2.302	14.638
Log_2_ fold change	−2.355	−2.028	−1.358
Adjusted *P*-value	0	0	0.006

**Table 3B:** Genes down regulated in one resistant strain only vs. MHco3(ISE).

**mRNA**	**HCOI02065400**	**HCOI00706800**	**HCOI02177200**	**HCOI01954200**	**HCOI00499800**
Predicted protein	Collagen	MOZ SAS containing	WD-40 repeat-like domain	ATPase domain	7 TM GPCR domain
Normalized expression MHco3(ISE)	48.3	131.55	133.42	368.48	21.84
Normalized expression MHco4(WRS)	35.6	105.816	178.11	115.2	10.97
Log_2_ fold change	−0.44	−0.314	−0.177	−1.677	−0.994
Adjusted *P*-value	0.144	0.335	0.413	0	0.023
Normalized expression MHco10(CAVR)	21.476	70.421	88.76	281.73	14.45
Log_2_ fold change	−1.169	−0.902	−0.594	−0.387	−0.595
Adjusted *P*-value	0	0.002	0.001	0.343	0.188

**Table 4A:** *C. elegans* homologs and RNAi phenotypes of genes down-regulated in both resistant strains.

**Hco gene**	***C. elegans* homolog**	**Predicted protein**	**Function**	**RNAi phenotype**
HCOI00084600	R06C1.4	Ortholog of human CSTF2 (Cleavage stimulation factor, 3′ pre-RNA, sub-unit 2)	Nucleic acid binding; embryonic development	None
HCOI01910900	None			
HCOI00821400	F22F7.7	Ortholog of human CHAC1 (gamma-glutamyl cyclotransferase)	Located to striated muscle- structural	None

**Table 4B:** *C. elegans* homologs and RNAi phenotypes of genes down-regulated in one resistant strain only.

**Hco gene**	***C. elegans* homolog**	**Predicted protein**	**Function**	**RNAi/mutant phenotype**
HCOI02065400	M110.1 (*col-76*)	Collagen	Cuticle	Unknown
HCOI00706800	R07B5.9 (*lsy-12*)	Histone acetyltransferase	DNA binding; required for neuronal differentiation; gonad formation, locomotion, etc.	Bagging, sick, protruding vulva, egg-laying defects
HCOI02177200	F49D11.10	Ortholog of human glycolipid transfer domain; WD repeat protein 75	Unknown	Unknown
HCOI01954200	F49C12.13 (*vha-17*)	Ortholog of vacuolar proton-translocating ATPase	Proton transport; embryo development, syncytial fusion, body morphogenesis. Localized to gut cells, gut granules, hypodermis	Apoptosis increased/ variant; embryonic lethal etc.
HCOI00499800	Y22D7AR (*ser-4*)	Ortholog of mammalian 5-HT1 serotonin receptor	Signal transduction; lifespan; negative regulator of locomotion; egg-laying	hyperactive, amplitude of sinusoidal movement variant, extended life span

**Figure 6:**
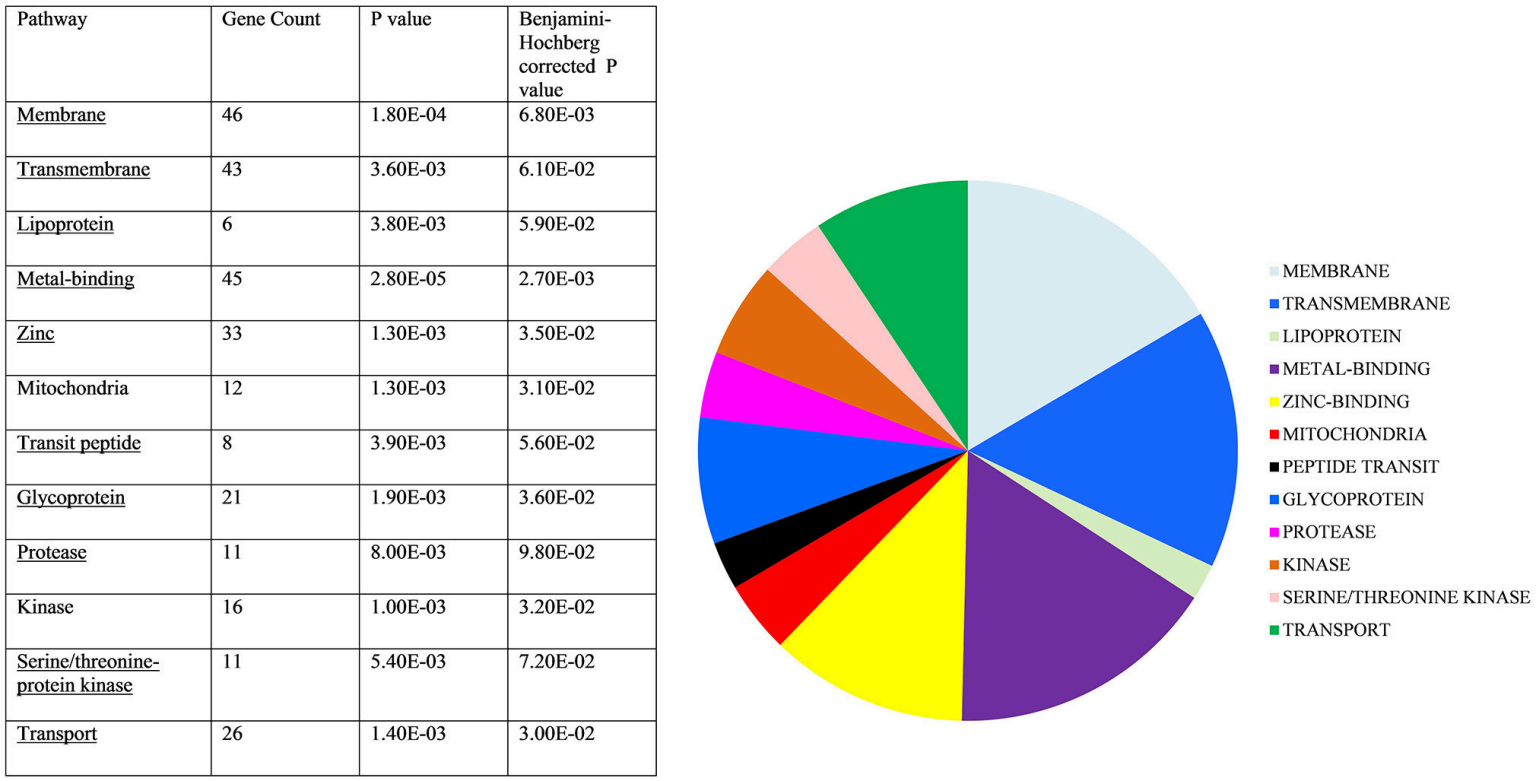
Target gene pathway analysis. *C. elegans* homologs of predicted *hco-miR-9551* targets were entered into DAVID Bioinformatics Database. Those significantly enriched are shown above. Statistically significant target gene pathways identified by DAVID (Benjamini-Hochberg score *p* < 0.05).

As the analysis described above relied upon the relatively stringent requirement for each mRNA target to be recognized by all three programmes, genes of interest could potentially be excluded. We addressed this issue by adopting a broader, overall view of the predicted targets to identify pathways enriched in the target gene data. The top 400 hits were selected from each of the three target prediction programmes, their orthologs identified in *C. elegans* and analyzed using the DAVID bioinformatic database. Allowing for genes that appeared in two or more lists and those which did not have orthologs in *C. elegans*, 485 WormBase identifiers were used to create a gene list, which was entered into the DAVID online tool. Functional annotation clusters were generated and significant pathways identified (Benjamini-Hochberg adjusted score *p* < 0.05, see Figure 6). Individual genes in each pathway are outlined in Table S4. Importantly, these included membrane/transmembrane associated genes, lipoproteins, metal-binding genes and transport-associated genes. Although P-glycoproteins are the classical drug transporters in nematodes and have been associated with drug resistance (Kerboeuf and Guegnard, 2011; Choi et al., 2017), these were not identified in the DAVID pathway analysis, suggesting involvement of a novel mechanism of resistance.

### Confirming predicted *hco-miR-9551* interactions

Three predicted *hco-miR-9551*/mRNA interactions from the transcriptomic analysis were further investigated by transfection of HEK293 cells in a dual luciferase assay. 3′ UTRs of HCOI00821400, HCOI00084600, and HCOI01910900 from *H. contortus* were cloned downstream of firefly luciferase and transiently transfected into HEK293 cells along with a plasmid containing *hco-miR-9551* in the forward or reverse direction. The CHAC-domain containing protein HCOI00821400 contained two putative *hco-miR-9551* binding sites in the 3′ UTR, while HCOI00084600 and HCOI01910900 contain one. This was determined using the PITA algorithm (Kertesz et al., 2007) using a minimum seed size of 8, allowing for a single G:U wobble and a single mismatch and results were refined by considering only those with PITA ΔΔG energies of < −7 (see Table 5).

**Table 5:** High confidence *hco-miR-9551* target sites determined using PITA algorithim.

**Gene**	**Position**	**Seed**	**ΔGduplex**	**ΔGopen**	**ΔΔG**
HCOI00821400	638	8:0:1	−20	−5.89	−14.10
HCOI00821400	41	8:0:1	−15.9	−8.89	−7.00
HCOI00084600	481	8:1:0	−21.7	−10.69	−11.00
HCOI01910900	162	8:0:0	−20	−8.08	−11.91

**Figure 7:**
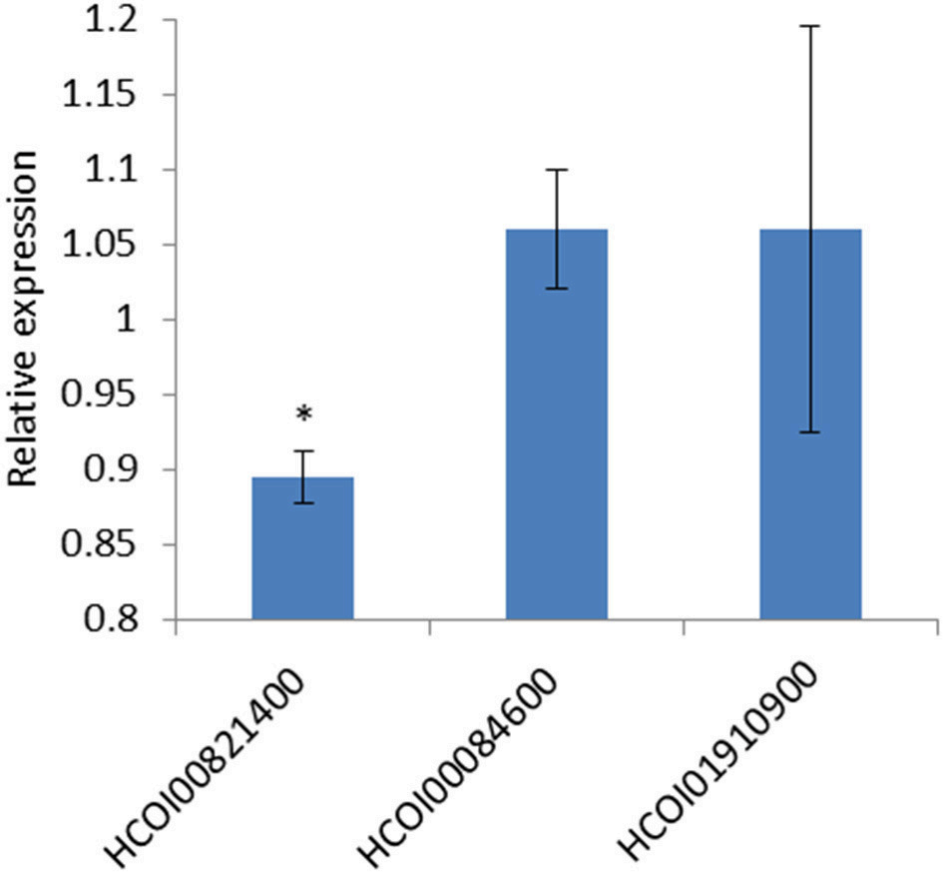
*hco-miR-9551* interacts with predicted target gene HCOI00821400 (CHAC-domain containing) 3′ UTR. Graph shows the repression of firefly luciferase in HEK293 cells transfected with the 3′ UTR of predicted target mRNA, HCOI00821400, but not with the 3′ UTR of HCOI00084600 or HCOI01910900. Data expressed as the mean ratio, ± standard deviation of three experiments comparing Firefly to Renilla luciferase signal in the presence of *hco-miR-9551* cloned in the forward orientation relative to the negative control (*hco-miR-9551* cloned in the reverse orientation) where the reverse is expressed as 1. *hco-miR-9551* and the 3′ UTR of HCOI00821400 resulted in a significant reduction in signal (*p* = 1.9E-5 *t* test). ^*^p < 0.05.

Levels of luciferase in the presence of the forward or reverse *hco-miR-9551* were compared. The 3′ UTR of HCOI00821400 showed a small (10.5%) but highly significant reduction (*p* = 1.9E-5) in expression in the presence of the forward *hco-miR-9551*, while there was no difference in expression for the 3′ UTRs of HCOI00084600 and HCOI01910900 when cells were transfected with the forward or reverse *hco-miR-9551*.The mean of three experiments comparing expression in the presence of the forward *hco-miR-9551* relative to the reverse is shown in Figure 7.

## Discussion

Understanding the mechanisms by which parasites develop drug resistance is important for improved diagnosis and for monitoring the spread of resistant alleles. While most studies on IVM resistance have focused on identifying mutations in presumed target sites, differences in the levels of expression of resistance-related genes have been observed. In this study, we investigated whether IVM resistance was associated with alterations in the expression of regulatory RNAs that could influence gene expression, as reported in some tumor cell models of drug resistance (Adam et al., 2009; Ahmad et al., 2015; Abdi et al., 2016). By microarray analysis, a single miRNA, *hco-miR-9551*, was found to be differentially expressed in all four IVM resistant strains compared to susceptible worms. This miRNA was most abundant in the two parental strains, MHco4(WRS) and MHco10(CAVR), and was also significantly upregulated in the backcross lines, MHco3/4.BC_4_ and MHco3/10.BC_4_. The IVM-resistant strains used in this study, MHco3(WRS) and MHco10(CAVR) derive from geographically distinct areas (South Africa and Australia, respectively) and are genetically divergent from each other. Consequently, one would predict that resistance in the two strains might have originated independently. The results obtained with both backcrosses are particularly compelling, as current estimates suggest that between 5 and 10% of the genome of the resistant parent is introgressed into the respective backcross (Rezansoff and Gilleard, unpublished). While the *hco-miR-9551* locus does not appear to be physically linked to a major introgressed region identified so far, we cannot rule out the possibility that it may be linked with shorter, as yet unidentified, introgressed sequences. *hco-miR-9551* appears to be restricted to clade V parasitic nematodes, a group containing many important pathogens of humans and animals in which anthelmintic resistance is widely observed. Importantly, *miR-9551* is also significantly upregulated in anthelmintic resistant *T. circumcincta*, the most prevalent parasitic nematode in UK sheep, in which multi-drug resistance is a major problem (Sargison et al., 2007). Taken together these data show that *miR-9551* expression is correlated with an anthelmintic resistance phenotype in two important parasitic nematodes.

Over 80% of one-to-one orthologs in *H. contortus* and *C. elegans* occur on the same chromosome (Laing et al., 2013) and by analogy with gene content in *C. elegans, hco-miR-9551* is predicted to reside on the X chromosome of *H. contortus*. A recent study demonstrated that in nematodes, the X chromosome is enriched for genes with female-biased expression (Albritton et al., 2014). In keeping with this finding, the expression of *hco-miR-9551* is enriched in female worms of the susceptible MHco3(ISE) isolate, as confirmed by analysis of different life cycle stages by microarray and qRT-PCR. Analysis of *hco-miR-9551* expression in male worms and L3 in all strains produced inconsistent results, likely related to the very low level of expression of *hco-miR-9551* in these stages. Sex-linked difference in susceptibility to IVM have been reported previously with female MHco10(CAVR) worms being more resistant than males (Le Jambre et al., 1995), but other non-sex-linked factors also play a role in drug resistance. The only other miRNA with significant homology *to hco-miR-9551* is *Drosophila melanogaster miR-30*8; a previous study showed that inhibition of this miRNA was associated with increased levels of apoptosis in embryos via de-repression of pro-apoptotic factors (Leaman et al., 2005). Whether the up-regulation of *hco-miR-9551* may have a similar embryo-protective role in the face of IVM exposure remains to be determined, but this could explain its sex-specific expression profile. In addition to its paralyzing effect on worms, IVM also affects embryogenesis in nematodes (Scott et al., 1991; Chavasse et al., 1992), but the precise mechanisms by which this effect is mediated remain largely unresolved.

Identifying differentially regulated miRNAs is only part of the story: understanding the potential role of miRNAs in drug resistance requires identification of the mRNA targets. We identified possible *hco-miR-9551* targets from a searchable database of predicted 3′ UTR sequences. Although it is now appreciated that miRNAs can bind elsewhere in a gene, including at sites in the open reading frame or 5′UTR (Zisoulis et al., 2010), the best-characterized binding sites are those in the 3′ UTR. To narrow down the large number of “hits,” we applied three separate programmes, each of which use different search parameters, and focused only on genes that were identified by all three and that had predicted overlapping binding sites in the 3′ UTR. 68 “high confidence” genes were identified in common among the three lists of putative targets significantly more than would be expected by chance (*p* < 0.001, Chi-square test). The expression of these 68 genes was then further analyzed using transcriptomic data from the susceptible, MHco3(ISE) and resistant parental strains, MHco4(WRS) and MHco10(CAVR) resulting in the identification of several genes containing *hco-miR-9551* binding sites that were down-regulated in the resistant strains.

Of the three genes down-regulated in both resistant strains one encoded a protein with a RNA recognition domain which, by analogy with *C. elegans*, is likely to be involved in polyadenylation of mRNA. The mammalian homolog, CSTF2, binds to GU rich elements in the 3′ UTR of mRNA and is reported to be over-expressed in some tumors (Deka et al., 2005). Of the other predicted targets of *hco-miR-9551* down-regulated in both resistant strains, one encodes a hypothetical protein and the other, a CHAC protein domain-containing protein. CHAC1 has gamma-glutamyl cyclotransferase (GGCT) activity, catalyzing the cleavage of glutathione to 5-oxy-proline and free amino acids. Down regulation of GGCT activity has previously been implicated in drug resistance in an adriamycin-resistant breast cancer cell line MCF-7 (MFC-7/ADR). In that study it was proposed that decreased activity of GGCT is necessary for MCF-7/ADR cells to maintain a high level of glutathione, which in turn exports adriamycin out of the cell (Wang et al., 2015). Conversely, when over expressed, CHAC1 can lead to the intracellular depletion of glutathione and the initiation of apoptosis (Wagstaff et al., 2012). The *H. contortus* CHAC1 is predicted to have a conserved GGCT domain and a reduction in CHAC1 protein could result in a tissue-specific increase in glutathione levels that may enhance detoxification pathways or protect against apoptosis by maintaining glutathione levels in drug resistant worms. Resistance can be manifest in multiple ways, other than direct effects on adult worm survival; for example, mechanisms that enhance embryo survival may also contribute to a resistance phenotype and may explain the female specific expression of *hco*-*miR-9551*. Interestingly, of the three genes down-regulated in resistant worms and predicted to be targets of *hco-miR-9551*, only *chac-1* is located on a scaffold likely to be within a region introgressed into the MHco3(ISE) genome from the resistant parents (Rezansoff and Gilleard, unpublished) and, significantly, this was the only 3′ UTR of three tested that showed an interaction with *hco-miR-9551* in the dual luciferase assay.

Of the genes down-regulated only in the MHco10(CAVR) IVM resistant strain, one is a predicted histone acetyltransferase. The *C. elegans* homolog of this gene, *lsy-12*, is important for neuronal differentiation and gonad formation. *lsy-12* mutant worms display a range of additional defects, including abnormalities in locomotion, egg-laying, and fecundity (Sarin et al., 2007, 2008). Other genes down-regulated in MHco10(CAVR) worms include a protein related to a human glycolipid transfer protein GLTPD2, which contains WD repeats, a type of protein that can be involved in pre-RNA processing. The two genes significantly down-regulated in MHco4(WRS) worms encode a vacuolar ATPase, which in *C. elegans*, is required for viability. *vha-17* is expressed in embryonic gut cells and is regulated by *pgp-2*, a member of the multi-drug resistance family of p-glycoproteins (Schroeder et al., 2007). The final gene encodes a protein with a predicted 7-transmembrane GPCR domain. The *C. elegans* homolog is *ser-4*, a mammalian serotonin receptor ortholog, which is expressed in a variety of neurons in the free-living worm (Tsalik et al., 2003).

While *miR-9551* was upregulated 6-fold in IVM-resistant *H. contortus* and up to 16-fold in resistant *T. circumcincta* it is difficult to predict the significance of these changes in a multicellular organism without additional information on where the miRNA is expressed within the worm, the number of potential mRNA targets, their expression levels and the strength of the interaction between miRNA and mRNA. However, much of the work on the role of miRNA in drug resistance has been carried out in tumor cells where there are many examples of miRNA expression changes of similar magnitude being involved in, or directly responsible for, chemoresistance (reviewed in Magee et al., 2015).

miRNAs frequently regulate genes within networks and so, in addition to identifying individual genes that may be targeted by *hco-miR-9551* to alter drug susceptibility, we also used pathway analysis to identify enrichment of specific functional networks. The major pathways included transport and membrane/transmembrane associated genes. In view of the hydrophobic nature of IVM, changes in the expression of receptors and channels involved in uptake and efflux of the drug across cell membranes are clearly relevant to resistance. Alternatively, we cannot rule out that hco-*miR-9551* upregulation is an indirect consequence of resistance, perhaps rebalancing membrane or transport networks responsible for resistance; these hypotheses will require further investigation. Nevertheless, our finding that a single miRNA is upregulated in the two parental and two backcrossed resistant lines of *H. contortus*, which originate from different geographical locations, as well as in resistant *T. circumcincta*, identifies a potentially novel mechanism of drug resistance in nematodes and contributes to understanding mechanisms of IVM resistance. These findings also have relevance in determining whether levels of resistance could be modulated and whether *hco-miR-9551* may represent a putative biomarker of IVM resistance in parasitic nematodes.

In conclusion, our study provides a novel paradigm by demonstrating that a regulatory RNA may play a role in this important phenomenon. While mutations in the targets of IVM, such as the GluCls, have been identified in many previous studies, other regulatory factors have not been examined. Non-coding RNAs are gaining prominence in other systems, both as drug targets and as mediators of resistance (Howe et al., 2015). As anthelmintic resistance continues its inexorable spread, looking beyond the protein targets of drugs to the regulatory mechanisms that influence resistance phenotypes could bear fruit.

## Author contributions

VG collected samples, carried out molecular lab work, participated in data analysis, and co-wrote the manuscript; KM collected samples and carried out molecular lab work; RL collected samples, provided bioinformatic support and critically read the manuscript; HG screened the 3′ UTR libraries and identified target mRNAs; NDM provided data from life span microarray experiments; AW advised on *H. contortus* miRNA experiments and critically read the manuscript; DB, AMo, and PS were responsible for maintaining all strains of *H. contortus* and carried out relevant infections; AR and JG provided RNAseq transcriptomic data, AMa constructed the *H. contortus* 3′ UTR database; ED collected samples, and ED and CB conceived and designed the study and co-wrote the manuscript. All authors gave their approval for publication.

### Conflict of interest statement

The authors declare that the research was conducted in the absence of any commercial or financial relationships that could be construed as a potential conflict of interest.
