# Supplementary material for: Increased Expression of a MicroRNA Correlates with Anthelmintic Resistance in Parasitic Nematodes
Source: Front Cell Infect Microbiol. 2017 Nov 6;7:452. doi: 10.3389/fcimb.2017.00452 (PMC5701612; doi:10.3389/fcimb.2017.00452)

S1 Figure. *H. contortus* scaffold_496 showing the position of miR-9551 precursor sequence on the forward strand and the two most closely linked genes on the reverse strand. The putative *C. elegans* orthologs for the other gene models on the scaffold are Y102A11A.6 (an ortholog of a human endoplasmic reticulum-Golgi intermediate compartment), Y102A11A.7 (a protein of unknown function), *acd-3* (a sodium channel non-voltage gated sub-unit), *egrh-1* (a zinc finger domain-containing protein that regulates oocyte maturation and ovulation), and *dpf-2* (a serine-type peptidase). One further gene of the eight has no *C. elegans* ortholog.

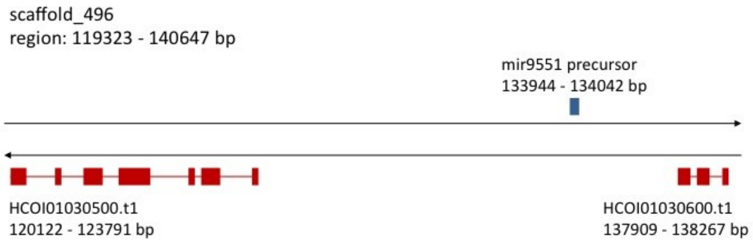

Supplement: Supplementary file 1 [file DataSheet1.PDF]
