# Supplementary material for: Increased Expression of a MicroRNA Correlates with Anthelmintic Resistance in Parasitic Nematodes
Source: Front Cell Infect Microbiol. 2017 Nov 6;7:452. doi: 10.3389/fcimb.2017.00452 (PMC5701612; doi:10.3389/fcimb.2017.00452)
Supplement: Supplementary file 2 [file DataSheet2.PDF]

**S1 Table. Pedigree of the backcrossed lines**

| Starting material as per Redman et al (2012) | Date | Sheep id number | Treatment IVM | L3 recovered | Adults |
|---|---|---|---|---|---|
| MHco3/4.$BC_4$ | Nov 2010 | 7500 | 0.2 mg/kg | Yes, used for infection of sheep 2348 | |
| MHco3/4.$BC_4$ S1 | July 2011 | 2348 | 0.2 mg/kg | Yes, used for infection of sheep 2353, 2277 & 2313 | |
| MHco3/4.$BC_4$ S2 | Sept 2011 | 2353, 2277, 2313 | 0.2 mg/Kg | Yes, frozen for future use | Used for array |
| | | | | | |
| MHco3/10.$BC_4$ | Nov 2010 | 3232 | 0.1 mg/kg | Yes, used for infection of sheep 2292 | |
| MHco3/10.$BC_4$ | Nov 2010 | 6555 | 0.2 mg/Kg | No | |
| MHco3/10.$BC_4$ S1 | July 2011 | 2292 | 0.2 mg/Kg | Yes, used for infection of sheep 2267, 2319 & 633J | |
| MHco3/10.$BC_4$ S2 | Sept 2011 | 2267, 2319, 633J | 0.2 mg/Kg | Yes, frozen for future use | Used for array |
