# Supplementary material for: Increased Expression of a MicroRNA Correlates with Anthelmintic Resistance in Parasitic Nematodes
Source: Front Cell Infect Microbiol. 2017 Nov 6;7:452. doi: 10.3389/fcimb.2017.00452 (PMC5701612; doi:10.3389/fcimb.2017.00452)
Supplement: Supplementary file 3 [file DataSheet3.PDF]

S2 Table. ***hco-miR-9551*** **homologs in clade V parasitic nematodes** *mismatch with hc-9551 underlined

**Species and contig**	**Co-ordinates**	**Sequence (Mature miRNA in uppercase)**	**Structure (using mfold http://mfold.rna.albany.edu/)**	**Initial ΔG**
Haemonchus contortus (PRJEB506) scaffold_496	133658:134333	TattagcatggcttagtagatctgttgctatctagtaaataggaTATCACAGCATTTTACTGAGCCctgctaaaa	10 20 30<br>ua u -| u c agu<br>uuagca ggcuuagu agaugcugu g uaucu a<br>aaucgu CCGAGUCA UUUACGACA C AUagg a<br>aa c U^ - U aua<br>70 60 50 40	-36.1
Ancylostoma caninum (PRJNA72585) ANCCANDFT_Contig1	3553682:3554352	tcttggcagtgctcggtaaatgctgttgctatccattctagcgaTATCACAGCATATTACTGAGCCcggct	10 20 30<br>ucuu agu - -----| ucc<br>ggc gcucgguaa augcugu ugcua \\<br>ucg CGAGUCAUU UACGACA gcgau a<br>---- gcC A CUAUa^ cuu<br>70 60 50 40	-27.8
Ancylostoma ceylanicum (PRJNA231479) Acey_s0241_scaf	161844:162514	tcttggcagtgctcggtaagtgctgttgctatccattcgagcgaTATCACAGCATGTTACTGAGCCcggct	10 20 30<br>ucuu agu -| u c cauu<br>ggc gcucgguaa gugcugu g uauc \\<br>ucg CGAGUCAUU UACGACA C AUag c<br>---- gcC G^ - U cgag<br>70 60 50 40	-26.7
Nippostrongylus brasiliensis (PRJEB511) NBR_scaffold0000115	30140:30807	tcagcagtgctcggtagacgctgttgctatccgtcagcgcgaTATCACAGCTTTTTACTGAGCCctgc	10 20 30<br>uca u c-| u c - u<br>gcag gcucgguaga gcugu g uauc cg c<br>cguc CGAGUCAUUU CGACA C AUag gc a<br>--- C UU^ - U c g<br>60 50 40	-31.4
Necator americanus (PRJNA72135) KI659148	241079:241739	gcttagtaaatgctgatgctatccgtttgagcgaTATCACAGCATCTTACTGAGCCcggct	10 20<br>------ -| a c c u<br>gcuuaguaa augcug ug uauc gu u<br>CGAGUCAUU UACGAC AC AUag cg g<br>ucggcC C^ - U - a<br>60 50 40 30	-23.1

<table>
<tr><td>Dictyocaulus viviparus (PRJEB5116) nDv.1.0.scaf00836</td><td>16786:17458</td><td>tttgctgtcctctgtaagtgctgctgctatccgaataggcgaTATCACAGCATTTTACGGAGCGcagccaaga</td><td>   10   20   30
uuu--   c   -| c c   cgaa
     gcugu cucugu aagugcug ug uauc   \
     cgacG GAGGCA UUUACGAC AC AUag   u
agaac   C   U^   - U   cgga
  70   60   50   40</td><td>-28.8</td></tr>
<tr><td>Teladorsagia circumcincta (PRJNA72569) TELCIRDFT_Contig74</td><td>245695:246367</td><td>attagcatggcttagtagatgctgttgttatccagtaagtacgaTATCACAGCATTTTACTGAGCCctgctga</td><td>   10   20   30
a   u   -| u u   cagu
 uuagca ggcuuagu agaugcugu g uauc   a
 agucgu CCGAGUCA UUUACGACA C AUag   a
-      c   U^   - U   caug
   70   60   50   40</td><td>-32.9</td></tr>
<tr><td>Strongylus vulgaris (PRJEB531) SVUK_scaffold0000023</td><td>10499:11169</td><td>tcttggcagtgctcagtaaatgctgttgctatctattctattgaTATCACAGCATCTTACTGAGCTcagct</td><td>   10   20   30
ucuu   agu   -| u c   uauu
    ggc   gcucaguaa augcugu g uauc   \
    ucg   CGAGUCAUU UACGACA C AUag   c
----   acU   C^   - U   uuau
    70   60   50   40</td><td>-28.1</td></tr>
<tr><td>Cylicostephanus goldi (PRJEB498) CGOC_contig0002454</td><td>1477:2145</td><td>ttggcagtgcttggtaaatgctgttgctatccgtactagcgaTATCACAGCATCTTACTGAGCCcagct</td><td>   10   20   30
uu   agu   ug   -| u c   c a
  ggc   gcu guaa augcugu g uauc gu c
  ucg   CGA CAUU UACGACA C AUag cg u
--   acC   GU   C^   - U   - a
            60   50   40</td><td>-24.7</td></tr>
<tr><td>Oesophagostomum dentatum (PRJNA72579) OESDENDFT_Contig1011</td><td>38031:38101</td><td>tctcagcagtgctcaggaagtgctgttgctatccgttcgagcgaTATCACAGCATCTTACTGAGCCcggct</td><td>   10   20   30
ucuc   agu   g   -| u c   c u
    agc   gcucag aag ugcugu g uauc gu c
    ucg   CGAGUC UUC ACGACA C AUag cg g
----   gcC   A   U^   - U   - a
    70   60   50   40</td><td>-26.8</td></tr>
</table>
