# Supplementary material for: Increased Expression of a MicroRNA Correlates with Anthelmintic Resistance in Parasitic Nematodes
Source: Front Cell Infect Microbiol. 2017 Nov 6;7:452. doi: 10.3389/fcimb.2017.00452 (PMC5701612; doi:10.3389/fcimb.2017.00452)
Supplement: Supplementary file 4 [file DataSheet4.PDF]

S3 Table. 68 'high confidence' genes with overlapping miR-9551 binding sites

Gene name	Gene name	Gene name
HCOI00859300	HCOI01623600	HCOI01345400
HCOI01324500	HCOI00834300	HCOI01361200
HCOI00074300	HCOI00864500	HCOI00042700
HCOI02129800	HCOI00907000	HCOI01682700
HCOI00169300	HCOI00921300	HCOI01737500
HCOI00170700	HCOI01429800	HCOI01845300
HCOI00170600	HCOI00708500	HCOI01398300
HCOI00205600	HCOI01612800	HCOI01910900
HCOI00214300	HCOI01652000	HCOI02028200
HCOI00222900	HCOI02103400	HCOI02065400
HCOI00256800	HCOI00821400	HCOI02083700
HCOI00331400	HCOI01585900	HCOI01754300
HCOI00145000	HCOI01698500	HCOI02150200
HCOI00341100	HCOI01854400	HCOI02177200
HCOI00350400	HCOI01954300	HCOI00749700
HCOI00355400	HCOI01954200	HCOI01226300
HCOI00370600	HCOI02133400	HCOI00970000
HCOI00375100	HCOI00459500	HCOI01287300
HCOI00499800	HCOI00459400	HCOI00706800
HCOI01385700	HCOI00496200	HCOI01397500
HCOI00584600	HCOI00523200	HCOI00O84600
HCOI00586100	HCOI00599900	HCOI00763100
HCOI00669200	HCOI00738800	
