# Supplementary material for: Increased Expression of a MicroRNA Correlates with Anthelmintic Resistance in Parasitic Nematodes
Source: Front Cell Infect Microbiol. 2017 Nov 6;7:452. doi: 10.3389/fcimb.2017.00452 (PMC5701612; doi:10.3389/fcimb.2017.00452)
Supplement: Supplementary file 5 [file DataSheet5.PDF]

S4 Table. Functional Annotation Clustering from DAVID. Gene lists associated with individual pathways.

Membrane associated genes

Gene > WormBase Gene ID	Gene > Gene Name	Gene > Sequence Name	Gene > Brief Description
**WBGene00000054**	acr-15	F25G6.4	A homolog of an alpha type nicotinic acetylcholine receptor subunit involved in the mediation of fast synaptic transmission at neuromuscular junctions.
**WBGene00000301**	cav-1	T13F2.8	cav-1 encodes one of two *C. elegans* caveolin orthologs; cav-1 is required for viability and Ras/MAP-kinase-dependent progression through the meiotic cell cycle; CAV-1 is expressed in the adult germ line and during embryonic development; CAV-1::GFP localizes to cortical granules that function to secrete chondroitin and mucin-like proteoglycans to the extracellular space following fertilization.
**WBGene00000390**	cdc-42	R07G3.1	cdc-42 encodes a RHO GTPase; cdc-42 controls polarity of both individual cells and developing embryos by regulating the localization of PAR proteins; cdc-42 is also required for regulation of receptor-mediated endocytosis; CDC-42 is widely expressed and localizes to a number of different locations, including the cell cortex, centrosomes, recycling endosomes, and at the time of hypodermal cell fusion, to hypodermal cell boundaries.
**WBGene00000464**	ceh-44	Y54F10AM.4	ceh-44 encodes at least three proteins via alternative splicing; the CEH-44A protein is an ortholog of the CUX class of CUT homeodomain proteins, expressed in early embryos (mostly anterior in the comma stage), in the larval nerve ring and the adult gonad; the CUX class includes Drosophila CUT, human CUTL1 and human CUTL2; ceh-44 is a complex gene, in which two alternatively spliced protein products completely lack the homeodomain and cut domain, and instead form a different protein, CASP; in vertebrates, this alternative splicing also occurs, while in Drosophila Casp as been lost; CASP proteins are also found in plants and fungi (though not associated with homeodomains); like its orthologs, CEH-44A has three cut domains upstream of the homeodomain; the cut domain is a DNA binding domain.
**WBGene00001039**	dnj-21	T19B4.4	This gene encodes a protein containing a DnaJ ('J') domain.
**WBGene00001334**	ero-1	Y105E8B.8	ero-1 encodes an endoplasmic reticulum oxidoreductase that affects ER-stress response and affects reactive oxygen species levels.
**WBGene00001365**	exc-4	Y105E8A.22	exc-4 encodes a highly conserved member of the chloride intracellular channel (CLIC) family of anion channels; EXC-4 is required during early stages of excretory cell tubulogenesis and appears also to play a role

			in vulval and seam cell development, which subsequently affects viability, fertility, and locomotion; EXC-4 is localized to the apical/lumenal membrane of the excretory cell and is also expressed in the hypodermis, vulva, and rectal gland cell.
**WBGene00001621**	glt-3	K08F4.4	glt-3 encodes an ortholog of glutamate/aspartate and neutral amino acid transporters.
**WBGene00002029**	hst-2	C34F6.4	hst-2 encodes the *C. elegans* ortholog of the heparan sulfate modifying enzyme 2O-sulfotransferase; by homology, HST-2 is predicted to function in heparan sulfate biosynthesis by catalyzing the chain-modifying sulfation of the C2 hydroxyl group of hexuronic acid; during development, hst-2 activity is required for normal body size, cell migration, and nervous system development; an hst-2::gfp reporter fusion is first expressed in embryos, continuing on through adulthood; expression is detected in many tissues, including the pharynx, hypodermis, muscles, vulva, and distal tip cells (DTCs) of the somatic gonad.
**WBGene00002151**	irk-3	K04G11.5	
**WBGene00002999**	lin-10	C09H6.2	lin-10 encodes a PDZ and PTB domain-containing protein that is homologous to mammalian Munc interacting proteins (Mint1, OMIM:602414) and is required for polarized protein localization; LIN-10 is required for proper localization of the LET-23 EGF receptor to the basolateral membrane of the vulval precursor cells and for proper postsynaptic localization of GLR-1, an AMPA-type glutamate receptor in interneurons; LIN-10 is detected in the cytoplasm, membrane, and at particularly high levels in the Golgi.
**WBGene00003570**	ncx-5	Y32F6B.2	ncx-5 encodes a putative 4Na[+]/1Ca[2+],1K[+] exchanger, orthologous to human SLC24A1-5 and paralogous to NCX-4; NCX-5 is predicted to export free cytoplasmic Ca[2+] with low affinity but high capacity, being complemented by low-capacity/high-affinity Ca[2+] ATPase pumps such as MCA-1/-3; NCX-5 has tandem Calx-alpha domains predicted to carry out ion transport; NCX-5 has no obvious function in mass RNAi assays.
**WBGene00003774**	nmr-1	F07F6.6	nmr-1 encodes an NMDA-type ionotropic glutamate receptor subunit that affects the duration of forward movement which is important during foraging behavior, and also affects osmotic avoidance; the slow kinetics typical of NMDA-dependent currents are likely important for its effect on forward movement.
**WBGene00004017**	phg-1	F27E5.4	
**WBGene00004048**	plx-2	K04B12.1	plx-2 encodes one of two *C. elegans* plexins, conserved transmembrane proteins that function as semaphorin receptors in both vertebrates and invertebrates; plx-2 activity is essential for proper male tail and epidermal morphogenesis and for normal axon guidance in a select group of neurons, including SDQL; genetic analyses indicate that plx-2 functions with mab-20/semaphorin-2a, efn-4/ephrin-4, and unc-129/TGF-beta to regulate male tail morphogenesis and with mab-20 and lad-2/L1CAM to mediate axon guidance; PLX-2 can bind MAB-20 and this interaction is enhanced in the presence of LAD-2; PLX-2::GFP reporters are expressed in a

			subset of neurons and epidermal cells, as well as a subset (1, 3, 5, and 7) of male sensory ray cell clusters.
**WBGene00004401**	rom-2	C48B4.2	
**WBGene00004760**	sel-2	F10F2.1	sel-2 encodes a PH, BEACH and WD40 domain-containing protein that is homologous to mammalian neurobeachin/BCL8B and LRBA (LPS-responsive, beige-like anchor protein); during development, SEL-2 functions to regulate endosomal traffic in polarized epithelial cells such as the vulval precursor cells (VPCs) and intestinal cells; specifically, in a subset of the VPCs, SEL-2 activity is required for proper levels and apical localization of LIN-12/Notch and for LET-23/EGFR downregulation; sel-2::yfp reporters are expressed in a number of cell types, including the VPCs and intestinal cells; a rescuing SEL-2::GFP reporter is expressed most strongly in the rectal epithelial cells and hypodermal seam cells where it localizes to the cytoplasm and the perinuclear region.
**WBGene00004793**	shw-3	R186.5	
**WBGene00004894**	sms-3	Y22D7AL.8	
**WBGene00006798**	unc-64	F56A8.7	unc-64 encodes syntaxin, a plasma membrane receptor for intracellular vesicles that is orthologous to vertebrate syntaxin 1A (OMIM:186590) and Drosophila Syx1A; UNC-64 is required for normal locomotion and possibly also for insulin secretion; as an essential component of the core synaptic vesicle fusion machinery, UNC-64 interacts with UNC-13, a diacylglycerol-binding protein, and SNB-1/synaptobrevin; UNC-64 trafficking from the endoplasmic reticulum to the plasma membrane is mediated by UNC-18, an SM (Sec1, Munc18) family member; unc-64 mutations can be suppressed by mutations in slo-1, a calcium-activated potassium channel; UNC-64 is expressed ubiquitously in the nervous system and in secretory cells such as the vulval uv1 cell and the excretory gland cells.
**WBGene00006869**	vab-2	Y37E11AR.6	The vab-2 gene encodes an ephrin molecule related to human ephrin B2 (OMIM:600527); VAB-2, expressed primarily in neuronal cells, is a ligand for the VAB-1 ephrin receptor and plays a role in embryonic cell movements, epidermal morphogenesis during later embryogenesis, and oocyte maturation; VAB-2 synergizes with PTP-3, a LAR-like receptor tyrosine phosphatase, to regulate morphogenesis.
**WBGene00007464**		C08H9.3	
**WBGene00007750**	syg-2	C26G2.1	syg-2 encodes a transmembrane protein that is a member of the immunoglobulin superfamily of proteins; during larval development, SYG-2 activity is required in vulval epithelial cells for proper synaptic specificity of the HSNL neuron; in regulating synapse formation, SYG-2 acts as a guidepost protein for the SYG-1 receptor that interacts with SYG-2 and acts within HSNL to regulate synaptic specificity; a SYG-2::GFP

			fusion protein is expressed in the primary vulval cell lineages beginning at the L3 larval stage, with expression increasing during the L4 stage and finally disappearing by adulthood; in embryos, SYG-2::GFP expression is detected in some head neurons and body wall muscles, the latter of which also express the reporter during the L1 and L2 larval stages.
**WBGene00007755**		C27A7.3	
**WBGene00008414**		D2030.4	D2030.4 encodes the *C. elegans* ortholog of the NDUFB7/B18 subunit of the mitochondrial NADH dehydrogenase (ubiquinone) complex (complex I).
**WBGene00008570**	kcnl-2	F08A10.1	
**WBGene00008800**		F14E5.2	
**WBGene00009164**	hrdl-1	F26E4.11	
**WBGene00009559**	mtx-1	F39B2.11	
**WBGene00009882**	vha-17	F49C12.13	vha-17 (aka 'fus-1') encodes an an ortholog of subunit e of the membrane-bound (V0) domain of vacuolar proton-translocating ATPase (V-ATPase); VHA-17, like VHA-1 and VHA-12, antagonizes EFF-1-mediated cell fusion in hypodermal cells; VHA-17 is expressed in gut cells of comma stage embryos, and in excretory cell and apical membranes of gut cells at later embryonic stages; VHA-17 is found in gut granules (a type of lysosome, expected to require acidification), and glo-1 mutant intestines fail to show punctate VHA-17; VHA-17 is required for viability, but this is at least somewhat genetically separable from VHA-17's antifusogenic activity.
**WBGene00012105**		T27F6.6	
**WBGene00013672**	catp-1	Y105E8A.12	catp-1 encodes an alpha subunit of the Na+/K+- and H+/K+-pump P-type ATPase family; catp-1 was identified in screens for animals resistant to developmental defects seen in larvae treated with the nicotinic agonist DMPP; genetic analyses indicate that catp-1 likely functions together with the let-60/Ras and daf-2/insulin-like receptor signaling pathways to regulate L2 larval developmental timing and dauer formation in a manner independent of its ATPase domain and thus, predicted transporter activity; a catp-1::gfp promoter fusion is expressed in hypodermal cells and in the excretory duct cell; hypodermal-specific catp-1(RNAi)

			confers partial resistance to DMPP, suggesting that hypodermal CATP-1 expression mediates DMPP toxicity.
**WBGene00014075**	dhhc-4	ZK757.4	
**WBGene00015165**		B0361.11	
**WBGene00015648**	orai-1	C09F5.2	
**WBGene00017261**	acl-6	F08F3.2	acl-6 encodes a mitochondrial glycerol-3-phosphate acyltransferase; acl-6 is predicted to play a role in triacylglycerol biosynthesis, and acl-6 expression is upregulated in animals during dauer larval development in response to direct exposure to daumone.
**WBGene00018044**		F35D11.3	
**WBGene00018181**		F38E1.9	
**WBGene00018418**		F44E2.4	
**WBGene00019300**	swt-1	K02D7.5	
**WBGene00019481**	cogc-6	K07C11.9	cogc-6 encodes an ortholog of mammalian COG-6, a subunit of lobe B of the conserved oligomeric Golgi complex (COGC); COGC-6 is weakly required for normal gonadal distal tip cell migration, a process that also requires seven other orthologs of COGC subunits; like other lobe B subunits in both *C. elegans* and *S. cerevisiae*, COGC-6 is only partially required for normal function, while lobe A subunits are strongly required in either worms or yeast.
**WBGene00019607**		K10B2.4	
**WBGene00019900**	vdac-1	R05G6.7	
**WBGene00020096**		R144.6	
**WBGene00022295**	cng-2	Y76B12C.1	
**WBGene00022580**	iglr-2	ZC262.3	

Transmembrane associated genes

Gene > WormBase Gene ID	Gene > Gene Name	Gene > Sequence Name	Gene > Brief Description
**WBGene00000054**	acr-15	F25G6.4	A homolog of an alpha type nicotinic acetylcholine receptor subunit involved in the mediation of fast synaptic transmission at neuromuscular junctions.
**WBGene00000301**	cav-1	T13F2.8	cav-1 encodes one of two *C. elegans* caveolin orthologs; cav-1 is required for viability and Ras/MAP-kinase-dependent progression through the meiotic cell cycle; CAV-1 is expressed in the adult germ line and during embryonic development; CAV-1::GFP localizes to cortical granules that function to secrete chondroitin and mucin-like proteoglycans to the extracellular space following fertilization.
**WBGene00000367**	cca-1	C54D2.5	cca-1 encodes a calcium channel alpha subunit that is homologous to vertebrate T-type calcium channel alpha 1 subunits; CCA-1 is required for regulation of pharyngeal pumping, specifically for the efficient initiation of action potentials in the pharynx in response to excitatory inputs; a CCA-1::GFP fusion protein is expressed strongly in pharyngeal muscle as well as in many neurons, including particular subsets of neurons in the head, pharynx, ventral nerve cord, and anal ganglia.
**WBGene00000464**	ceh-44	Y54F10AM.4	ceh-44 encodes at least three proteins via alternative splicing; the CEH-44A protein is an ortholog of the CUX class of CUT homeodomain proteins, expressed in early embryos (mostly anterior in the comma stage), in the larval nerve ring and the adult gonad; the CUX class includes Drosophila CUT, human CUTL1 and human CUTL2; ceh-44 is a complex gene, in which two alternatively spliced protein products completely lack the homeodomain and cut domain, and instead form a different protein, CASP; in vertebrates, this alternative splicing also occurs, while in Drosophila Casp as been lost; CASP proteins are also found in plants and fungi (though not associated with homeodomains); like its orthologs, CEH-44A has three cut domains upstream of the homeodomain; the cut domain is a DNA binding domain.
**WBGene00000524**	clc-3	ZK563.4	clc-3 encodes a claudin homolog that may regulate ion channels; CLC-3 is worm-specific, with a highly divergent sequence weakly similar to mammalian voltage-dependent calcium channel gamma subunits that are known or suspected to prevent epilepsy in vivo (e.g., stargazin; MGI:1316660); claudins are integral membrane proteins with four transmembrane sequences that are found in mammalian tight junctions (TJs), induce TJs when transgenically expressed in cells normally lacking them, and can mediate the specific conductance of of specific ions (e.g., magnesium or calcium) through TJs while blocking the flow of water.
**WBGene00001039**	dnj-21	T19B4.4	This gene encodes a protein containing a DnaJ ('J') domain.

**WBGene00001365**	exc-4	Y105E8A.22	exc-4 encodes a highly conserved member of the chloride intracellular channel (CLIC) family of anion channels; EXC-4 is required during early stages of excretory cell tubulogenesis and appears also to play a role in vulval and seam cell development, which subsequently affects viability, fertility, and locomotion; EXC-4 is localized to the apical/lumenal membrane of the excretory cell and is also expressed in the hypodermis, vulva, and rectal gland cell.
**WBGene00001621**	glt-3	K08F4.4	glt-3 encodes an ortholog of glutamate/aspartate and neutral amino acid transporters.
**WBGene00002029**	hst-2	C34F6.4	hst-2 encodes the *C. elegans* ortholog of the heparan sulfate modifying enzyme 2O-sulfotransferase; by homology, HST-2 is predicted to function in heparan sulfate biosynthesis by catalyzing the chain-modifying sulfation of the C2 hydroxyl group of hexuronic acid; during development, hst-2 activity is required for normal body size, cell migration, and nervous system development; an hst-2::gfp reporter fusion is first expressed in embryos, continuing on through adulthood; expression is detected in many tissues, including the pharynx, hypodermis, muscles, vulva, and distal tip cells (DTCs) of the somatic gonad.
**WBGene00002151**	irk-3	K04G11.5	
**WBGene00002173**	itr-1	F33D4.2	itr-1 encodes a putative inositol (1,4,5) trisphosphate receptor that affects the defecation cycle and pharyngeal pumping, and also affects ovulation in a pathway downstream of LET-23; interacts with UNC-54 in vivo, and is expressed in the adult intestine, pharynx, excretory cell, germ line, and spermatheca, with limited neuronal expression.
**WBGene00003570**	ncx-5	Y32F6B.2	ncx-5 encodes a putative 4Na[+]/1Ca[2+],1K[+] exchanger, orthologous to human SLC24A1-5 and paralogous to NCX-4; NCX-5 is predicted to export free cytoplasmic Ca[2+] with low affinity but high capacity, being complemented by low-capacity/high-affinity Ca[2+] ATPase pumps such as MCA-1/-3; NCX-5 has tandem Calx-alpha domains predicted to carry out ion transport; NCX-5 has no obvious function in mass RNAi assays.
**WBGene00003774**	nmr-1	F07F6.6	nmr-1 encodes an NMDA-type ionotropic glutamate receptor subunit that affects the duration of forward movement which is important during foraging behavior, and also affects osmotic avoidance; the slow kinetics typical of NMDA-dependent currents are likely important for its effect on forward movement.
**WBGene00004048**	plx-2	K04B12.1	plx-2 encodes one of two *C. elegans* plexins, conserved transmembrane proteins that function as semaphorin receptors in both vertebrates and invertebrates; plx-2 activity is essential for proper male tail and epidermal morphogenesis and for normal axon guidance in a select group of neurons, including SDQL; genetic analyses indicate that plx-2 functions with mab-20/semaphorin-2a, efn-4/ephrin-4, and unc-129/TGF-beta to regulate male tail morphogenesis and with mab-20 and lad-2/L1CAM to mediate axon guidance; PLX-2 can bind MAB-20 and this interaction is enhanced in the presence of LAD-2; PLX-2::GFP reporters are expressed in a subset of neurons and epidermal cells, as well as a subset (1, 3, 5, and 7) of male sensory ray cell clusters.
**WBGene00004401**	rom-2	C48B4.2	

**WBGene00004793**	shw-3	R186.5	
**WBGene00004894**	sms-3	Y22D7AL.8	
**WBGene00006798**	unc-64	F56A8.7	unc-64 encodes syntaxin, a plasma membrane receptor for intracellular vesicles that is orthologous to vertebrate syntaxin 1A (OMIM:186590) and Drosophila Syx1A; UNC-64 is required for normal locomotion and possibly also for insulin secretion; as an essential component of the core synaptic vesicle fusion machinery, UNC-64 interacts with UNC-13, a diacylglycerol-binding protein, and SNB-1/synaptobrevin; UNC-64 trafficking from the endoplasmic reticulum to the plasma membrane is mediated by UNC-18, an SM (Sec1, Munc18) family member; unc-64 mutations can be suppressed by mutations in slo-1, a calcium-activated potassium channel; UNC-64 is expressed ubiquitously in the nervous system and in secretory cells such as the vulval uv1 cell and the excretory gland cells.
**WBGene00007464**		C08H9.3	
**WBGene00007755**		C27A7.3	
**WBGene00008570**	kcnl-2	F08A10.1	
**WBGene00008800**		F14E5.2	
**WBGene00009164**	hrdl-1	F26E4.11	
**WBGene00009559**	mtx-1	F39B2.11	
**WBGene00009882**	vha-17	F49C12.13	vha-17 (aka 'fus-1') encodes an an ortholog of subunit e of the membrane-bound (V0) domain of vacuolar proton-translocating ATPase (V-ATPase); VHA-17, like VHA-1 and VHA-12, antagonizes EFF-1-mediated cell fusion in hypodermal cells; VHA-17 is expressed in gut cells of comma stage embryos, and in excretory cell and apical membranes of gut cells at later embryonic stages; VHA-17 is found in gut granules (a type of lysosome, expected to require acidification), and glo-1 mutant intestines fail to show punctate VHA-17; VHA-17 is required for viability, but this is at least somewhat genetically separable from VHA-17's

			antifusogenic activity.
**WBGene00012105**		T27F6.6	
**WBGene00013672**	catp-1	Y105E8A.12	catp-1 encodes an alpha subunit of the Na+/K+- and H+/K+-pump P-type ATPase family; catp-1 was identified in screens for animals resistant to developmental defects seen in larvae treated with the nicotinic agonist DMPP; genetic analyses indicate that catp-1 likely functions together with the let-60/Ras and daf-2/insulin-like receptor signaling pathways to regulate L2 larval developmental timing and dauer formation in a manner independent of its ATPase domain and thus, predicted transporter activity; a catp-1::gfp promoter fusion is expressed in hypodermal cells and in the excretory duct cell; hypodermal-specific catp-1(RNAi) confers partial resistance to DMPP, suggesting that hypodermal CATP-1 expression mediates DMPP toxicity.
**WBGene00013782**	npr-32	Y116A8B.5	
**WBGene00014075**	dhhc-4	ZK757.4	
**WBGene00015165**		B0361.11	
**WBGene00015648**	orai-1	C09F5.2	
**WBGene00017261**	acl-6	F08F3.2	acl-6 encodes a mitochondrial glycerol-3-phosphate acyltransferase; acl-6 is predicted to play a role in triacylglycerol biosynthesis, and acl-6 expression is upregulated in animals during dauer larval development in response to direct exposure to daumone.
**WBGene00018044**		F35D11.3	
**WBGene00018181**		F38E1.9	
**WBGene00018418**		F44E2.4	
**WBGene00019300**	swt-1	K02D7.5	
**WBGene00019607**		K10B2.4	
**WBGene00019900**	vdac-1	R05G6.7	

**WBGene00020069**		R13H7.2	
**WBGene00020096**		R144.6	
**WBGene00022014**		Y61A9LA.1	
**WBGene00022295**	cng-2	Y76B12C.1	
**WBGene00022580**	iglr-2	ZC262.3	

Lipoprotein related genes

Gene > WormBase Gene ID	Gene > Gene Name	Gene > Sequence Name	Gene > Brief Description
**WBGene00000301**	cav-1	T13F2.8	cav-1 encodes one of two *C. elegans* caveolin orthologs; cav-1 is required for viability and Ras/MAP-kinase-dependent progression through the meiotic cell cycle; CAV-1 is expressed in the adult germ line and during embryonic development; CAV-1::GFP localizes to cortical granules that function to secrete chondroitin and mucin-like proteoglycans to the extracellular space following fertilization.
**WBGene00000390**	cdc-42	R07G3.1	cdc-42 encodes a RHO GTPase; cdc-42 controls polarity of both individual cells and developing embryos by regulating the localization of PAR proteins; cdc-42 is also required for regulation of receptor-mediated endocytosis; CDC-42 is widely expressed and localizes to a number of different locations, including the cell cortex, centrosomes, recycling endosomes, and at the time of hypodermal cell fusion, to hypodermal cell boundaries.
**WBGene00001669**	gpa-7	R10H10.5	gpa-7 encodes a member of the G protein alpha subunit family of heterotrimeric GTPases that affects egg laying and response to water- soluble odorants; it is expressed in excitable cells.
**WBGene00001672**	gpa-10	C55H1.2	gpa-10 encodes a member of the G protein alpha subunit family of heterotrimeric GTPases; it is expressed in ADF, ASI, ASJ, ALN, CAN, LUA, and the spermatheca.
**WBGene00004017**	phg-1	F27E5.4	
**WBGene00004201**	prx-19	F54F2.8	prx-19 is orthologous to the human gene PEROXISOMAL FARNESYLATED PROTEIN (PXF; OMIM:600279), which when mutated leads to peroxisome biogenesis (Zellweger) syndrome of complementation group J.

Metal binding associated genes

Gene > WormBase Gene ID	Gene > Gene Name	Gene > Sequence Name	Gene > Brief Description
**WBGene00000101**	aka-1	D1022.7	aka-1 encodes an A kinase anchor protein that has mutiple isoforms, all of which have a zinc-finger domain of the FYVE type; interacts with KIN-2 in vitro.
**WBGene00000102**	akt-1	C12D8.10	akt-1 encodes an ortholog of the serine/threonine kinase Akt/PKB; akt-1 genetically interacts with the insulin signaling pathway and functions to regulate such processes as dauer larval development and salt chemotaxis learning; AKT-1 binds calmodulin in vitro in a calcium-dependent manner; an AKT-1::GFP fusion protein is widely expressed beginning in late stage embryos and continuing through adulthood; expression is seen in head, tail, and dorsal and ventral cord neurons, with additional expression seen in other cells including those of the pharynx, hypodermis, intestine, and spermatheca; two alleles of akt-1 (sa573 and sa700) have a Daf-c mutant phenotype at 27 degrees C (Hid phenotype).
**WBGene00000366**	cbp-1	R10E11.1	cbp-1 encodes a homolog of the mammalian transcriptional cofactors CBP (OMIM:600140) and p300 (E1A-BINDING PROTEIN, 300-KD; OMIM:602700) that have been shown to possess histone acetyltransferase activity, and which, when mutated, lead to Rubinstein-Taybi syndrome (OMIM:180849) and colorectal cancer (OMIM:114500); at least one splicing form of CBP-1 exhibits histone acetyltransferase (HAT) activity in vitro and has a glutamine/asparagine-rich domain; CBP-1 is required during embryogenesis for differentiation of all non-neuronal somatic cell types; CBP-1 is expressed very early in embryogenesis, suggesting that it may interact with maternally provided transcription factors, such as SKN-1, to specific developmental fates.
**WBGene00000787**	cps-6	C41D11.8	cps-6 encodes an ortholog of human mitochondrial endonuclease G (EndoG) that promotes apoptosis, and is required to degrade nicked (TUNEL-positive) DNA in apoptotic cells; transgenic CPS-6 is localized to mitochondria, but transgenic CPS-6 lacking a mitochondrial localization sequence is found in nuclei, and nuclei may be the in vivo target of CPS-6 after its activation by CED-3; CPS-6 has magnesium-dependent nuclease activity in vitro and can degrade both single- and double-stranded DNA, as well as single-stranded RNA; CPS-6 also preferentially binds G-tract DNA in vitro; cps-6(sm116) and cps-6(RNAi) animals have delayed CED-3-induced apoptosis, and cps-6(sm116) suppresses a constitutively active ced-3 transgene; cps-6(sm116) can be transgenically rescued by mouse EndoG; CPS-6 binds WAH-1 (an apoptosis-inducing factor ortholog) in vitro, WAH-1 binding enhances CPS-6's endonuclease activity, and constitutive transgenic coexpression of cps-6 with wah-1 induces cell death not seen with constitutive expression of either cps-6 or wah-1 alone; CPS-6 binds CRN-1 (a flap endonuclease ortholog) in vitro, and may form a large complex in vivo with CRN-1, CRN-4, CRN-5, CYP-13, and WAH-1; CRN-1 enhances CPS-6's endonuclease activity in vitro, CPS-6 enhances CRN-1's gap-dependent endonuclease and 5'-3' exonuclease activities, and cps-6 is

			required for excess cell deaths induced by a crn-1 transgene.
**WBGene00000960**	dgk-3	F54G8.2	dgk-3 encodes a diacylglycerol kinase that is the *C. elegans* ortholog of mammalian DGK-beta; dgk-3 activity is required for regulation of long-term thermotactic behavioral plasticity and for regulation of olfactory adaptation; large-scale expression studies have reported dgk-3 expression in head neurons, the intestine, and the pharyngeal lumen, while expression profiling indicates that dgk-3 is expressed in the AFD thermosensory neurons as well as a small number of additional sensory neurons.
**WBGene00001412**	fem-2	T19C3.8	fem-2 encodes a protein phosphatase 2C; a CB5161 ortholog of fem-2, Cbn-fem-2, has been isolated from the sibling Caenorhabditis species CB5161.
**WBGene00001650**	gon-1	F25H8.3	gon-1 encodes a functional metalloprotease that defines a new sub-family of secreted proteases known as MPT (metalloprotease with thrombospondin type 1 repeats); the other two members of this family are the bovine procollagen I N-protease (PINP) and the murine enzyme ADAMTS-1; gon-1 is essential for hermaphrodite gonadal morphogenesis and sequence homology with other metalloproteases suggests that it functions by remodeling the extracellular matrix; gon-1 is also required for protein transport from the endoplasmic reticulum (ER) to the Golgi, a function dependent on its C-terminal GON domain; GON-1 is expressed at high levels within the gonadal distal tip cell during migration and also in body wall muscle cells; in the distal tip cell, reporter fusion constructs expressing the GON domain localize to the endoplasmic reticulum, suggesting that GON-1 functions both intra- and extracellularly.
**WBGene00003002**	lin-13	C03B8.4	lin-13 encodes a large (2248-residue) nuclear protein with multiple zinc fingers of the C2H2 class and a LXCXE retinoblastoma protein-binding motif, that is required for survival through larval development and for negative regulation of vulval fates during postembryonic development.
**WBGene00003533**	nas-14	F09E8.6	nas-14 encodes an astacin-like metalloprotease; large-scale expression studies reveal that a nas-14::GFP promoter fusion is expressed in the pharynx.
**WBGene00003548**	nas-30	Y95B8A.1	nas-30 encodes an astacin family zinc metalloprotease with a single EGF domain and a CUB domain; nas-30 is expressed in the intestine and hypodermis.
**WBGene00003551**	nas-33	K04E7.3	nas-33 encodes an astacin family zinc metalloprotease; nas-33 is expressed in the intestine and hypodermis.
**WBGene00003602**	nhr-3	H01A20.1	nhr-3 encodes a member of the superfamily of nuclear receptors which is one of the most abundant class of transcriptional regulators; nuclear receptors have a well conserved DNA binding domain and a less conserved C-terminal ligand binding domain; nhr-3 has been identified and characterised as a gene affected by ethanol exposure in a microarray analysis of all *C. elegans* ORFs.
**WBGene00003628**	nhr-35	C07A12.3	
**WBGene00004319**	rbr-2	ZK593.4	rbr-2 encodes a histone H3 lysine 4 (H3K4) demethylase orthologous to budding yeast Jhd2p, Drosophila LID, and human JARID1A (OMIM:180202), JARID1B (OMIM:605393), JARID1C OMIM:314690, mutated

			in mental retardation), and JARID1D (OMIM:426000); RBR-2 regulates genome-wide levels of H3K4 trimethylation and is required in the germ line for normally short lifespan; recombinant His-RBR-2 specifically demethylates H3K4me3/me2 in vitro; rbr-2 is also required for reliable vulval development, and interacts genetically with CLK-2, GLP-1, LIN-15, and SEM-5; rbr-2(tm1231) homozygotes display excess trimethylated H3K4 (H3K4me3) and erratic vulval development (either vulvaless or multivulva); rbr-2(RNAi) animals show extended lifespans, a strong synthetic multivulva and H3K4me3 phenotype in a lin-15(n765ts) mutant background at permissive temperature, and abnormally slow growth with clk-2(mn159), glp-1(or178), or sem-5(n2019) mutant backgrounds; an RBR-2::GFP fusion protein is widely expressed and localizes to nuclei.
**WBGene00006648**	ttb-1	W03F9.5	
**WBGene00009006**		F21D5.1	F21D5.1 encodes a putative phosphoacetylglucosamine mutase orthologous to human PGM3 (OMIM:172100); F21D5.1 is thought to catalyse the third step of the hexosamine pathway to UDP-N-acetylglucosamine or UDP-N-acetylgalactosamine; F21D5.1 transcripts are enriched during oogenesis; F21D5.1(RNAi) animals have an osmotically-sensitive embryonic lethal phenotype, presumably because of defects in chitin and eggshell synthesis.
**WBGene00009164**	hrdl-1	F26E4.11	
**WBGene00009647**		F43C1.1	
**WBGene00010155**		F56F3.4	
**WBGene00010397**	suox-1	H13N06.4	suox-1 encodes a sulfite oxidase orthologous to human SUOX (SUOX; OMIM:602216), which when mutated leads to disease.
**WBGene00010892**	dhhc-6	M18.8	
**WBGene00010923**	rle-1	M142.6	
**WBGene00010988**	metr-1	R03D7.1	R03D7.1 is orthologous to the human gene METHIONINE SYNTHASE (MTR; OMIM:156570), which when mutated leads to disease.
**WBGene00011004**		R04B5.6	R04B5.6 encodes one of two *C. elegans* sorbitol dehydrogense orthologs; by homology the product of R04B5.6 is predicted to catalyze the reversible oxidation of sorbitol to fructose in the presence of NAD+; in the embryo, an R04B5.6::gfp fusion is expressed in pharyngeal cells and head neurons.

**WBGene00012105**		T27F6.6	
**WBGene00012606**		Y38F1A.2	
**WBGene00012796**		Y43F4A.1	
**WBGene00013405**		Y63D3A.4	
**WBGene00013532**		Y73F8A.27	
**WBGene00014019**		ZK632.12	ZK632.12 encodes one of 12 *C. elegans* FYVE-domain containing proteins and is orthologous to mammalian Phafin2; as loss of ZK632.12 activity via RNAi screens results in no obvious defects, the precise role of ZK632.12 in *C. elegans* development and/or behavior is not yet known.
**WBGene00014030**	glb-1	ZK637.13	glb-1 encodes a globin; glb-1 transcription is uninduced by anoxia, but repressed by DAF-2 signalling in a DAF-16 dependent manner; glb-1 is expressed in distal germline, late embryonic cells that are probably hypodermal precursors, and young adults; glb-1 transcription is lower in L3 larvae than in adults, while in adults it is higher than that of most other globins; glb-1 has no obvious function in mass RNAi assays.
**WBGene00014075**	dhhc-4	ZK757.4	
**WBGene00014202**	mmcm-1	ZK1058.1	mmcm-1 encodes an ortholog of human methylmalonyl-CoA mutase (MUT, mutated in methylmalonic acidemia; OMIM:609058); MMCM-1 enzyme, in vitro, kinetically resembles its human ortholog; mmcm-1 deletion mutants incorporate abnormally low levels of 1-[(14)C]-propionate into proteins; mmcm-1(RNAi) and mmcm-1 deletion mutant animals excrete abnormally high levels of methylmalonic acid into their culture medium when challenged with propionic acid; mmcm-1, in a lentiviral transgene, can partially rescue the mutant phenotype of human mut(o) fibroblasts; these data are consistent with the hypothesis that MMCM-1 participates in the conversion of propionyl-CoA to succinyl-CoA.
**WBGene00016184**	glb-9	C28F5.2	glb-9 encodes a globin with no obvious function in mass RNAi assays.
**WBGene00016197**	pxl-1	C28H8.6	pxl-1 encodes proteins containing N-terminal LD motifs and C-terminal LIM domains that is the *C. elegans* ortholog of vertebrate paxillin; pxl-1 is required for organized myofilament structure in the pharynx and pharyngeal muscle contraction, and hence feeding and viability past the first larval stage; PXL-1 is expressed in body wall and pharyngeal muscle, as well as pharyngeal epithelial cells; PXL-1 localizes to sites of actin attachment, namely dense bodies, adhesion plaques, and M-lines in body wall muscle, and podosome-like structures in pharyngeal muscle; pxl-1 expression in pharyngeal muscle and marginal cells is sufficient to rescue the pharyngeal muscle defects and lethality of a pxl-1 mutant; PXL-1 physically interacts with muscle proteins DEB-1, UIG-1, LIM-8, UNC-96, UNC-95, and HUM-6.
**WBGene00017342**		F10D7.5	F10D7.5 encodes an ortholog of Drosophila NEURALIZED, and thus may participate in GLP-1/LIN-12

			signalling; the promoter or 5' protein-coding regions of F10D7.5 are bound by DAF-16 in vivo, and F10D7.5 expression drops by roughly one-half in daf-16(mu86) mutants.
**WBGene00017644**	exc-9	F20D12.5	exc-9 encodes one of two *C. elegans* small LIM domain-containing proteins related to the mammalian CRIP (Cysteine-Rich Intestinal Protein) family of proteins; in *C. elegans*, exc-9 activity is required cell autonomously for proper organization of the apical cytoskeleton of the excretory canal cell, which is essential for maintaining the diameter of the excretory canal tubules; in addition, exc-9 is required for proper morphogenesis of the hermaphrodite and male tails; genetic analyses indicated that exc-9 likely functions upstream of exc-5, which encodes a guanine-nucleotide exchange factor, and downstream of exc-2 and sma-1, in regulating canal cell morphology; an EXC-9::GFP fusion protein is expressed in a number of cell types including the excretory canal cell, the tail spike, the uterine seam cell and anchor cell, the intestine, ALM and PLN neurons, and the nerve ring; in males, EXC-9::GFP expression is seen in a few tail neurons and weakly throughout the tail during larval development; EXC-9::GFP localizes to the cytoplasm; in yeast two-hybrid assays, EXC-9 interacts with CSN-5, a subunit of the COP9 signalosome likely involved in ubiquitin-mediated protein degradation.
**WBGene00017904**	lim-8	F28F5.3	lim-8 encodes proteins containing one PDZ and one LIM domain; in vitro binding and yeast two-hybrid assays indicate that LIM-8 can physically interact with myosin heavy chain A (MYO-3) as well as with UNC-96 and UNC-97, suggesting that LIM-8 is part of a structural component that links membrane attachment proteins to myosin thick filaments; lim-8(RNAi) in a hypersensitive rrf-3 background results in partially penetrant paralysis at mid-larval stages of development; a lim-8::gfp promoter fusion is expressed in pharyngeal and body wall muscles, as well as in vulva, spermathecae, anal sphincter and depressor muscles, head neurons, gonadal sheath, and the excretory canal; staining with LIM-8 antibodies reveals that in body wall muscle LIM-8 localizes, at least partially, to M-lines, around which myosin thick filaments are organized.
**WBGene00018966**		F56D2.5	
**WBGene00020496**	spat-3	T13H2.5	spat-3 encodes, by transcription from alternative promoters, two large (1092- and 2471-residues) proteins; while residues 162-228 of the larger SPAT-3 protein encode a Ring1 type of zinc-finger domain (like that seen in SEX COMBS EXTRA, an E3 ubiquitin-protein ligase RING1 protein of Drosophila melanogaster), the bulk of SPAT-3 has no obvious similarities to other proteins; consistent with its predicted role as a PRC1 component, spat-3 activity is required in vivo for histone H2A ubiquitination; spat-3 is also required for proper positioning of Ray 1 in the male tail, neuronal migration and axon extension, vulval development, and fully wild-type brood sizes; SPAT-3 is also specifically required for PAR protein-dependent cell-polarity, and this requirement is independent of PAR-2 activity; as with par-3, par-6, pkc-3, and cdc-42, inactivation of spat-3 (by RNAi or mutation) strongly suppresses the embryonic lethality of par-2(it5ts) at restrictive temperature; as with nos-3, spat-3 also suppresses par-2(lw32), a strong loss-of-function allele; while spat-3 par-2 double mutant embryos require PAR-1 for viability, PAR-1 does not regain posterior cortical

			localization in these embryos, but is instead diffused through their cytoplasm.
**WBGene00020694**	dhhc-11	T22E7.2	
**WBGene00021347**	rpb-10	Y37E3.3	Y37E3.3 encodes the *C. elegans* ortholog of the RNA polymerase II Rbp10 subunit; loss of Y37E3.3 activity via large-scale RNAi results in early embryonic lethality.
**WBGene00021924**		Y55F3AM.6	
**WBGene00022794**	snu-23	ZK686.4	
**WBGene00044071**	dhhc-14	D2021.2	

Zinc binding associated genes

**Gene > WormBase Gene ID**	**Gene > Gene Name**	**Gene > Sequence Name**	**Gene > Brief Description**
**WBGene00000101**	aka-1	D1022.7	aka-1 encodes an A kinase anchor protein that has mutiple isoforms, all of which have a zinc-finger domain of the FYVE type; interacts with KIN-2 in vitro.
**WBGene00000366**	cbp-1	R10E11.1	cbp-1 encodes a homolog of the mammalian transcriptional cofactors CBP (OMIM:600140) and p300 (E1A-BINDING PROTEIN, 300-KD; OMIM:602700) that have been shown to possess histone acetyltransferase activity, and which, when mutated, lead to Rubinstein-Taybi syndrome (OMIM:180849) and colorectal cancer (OMIM:114500); at least one splicing form of CBP-1 exhibits histone acetyltransferase (HAT) activity in vitro and has a glutamine/asparagine-rich domain; CBP-1 is required during embryogenesis for differentiation of all non-neuronal somatic cell types; CBP-1 is expressed very early in embryogenesis, suggesting that it may interact with maternally provided transcription factors, such as SKN-1, to specific developmental fates.
**WBGene00000960**	dgk-3	F54G8.2	dgk-3 encodes a diacylglycerol kinase that is the *C. elegans* ortholog of mammalian DGK-beta; dgk-3 activity is required for regulation of long-term thermotactic behavioral plasticity and for regulation of olfactory adaptation; large-scale expression studies have reported dgk-3 expression in head neurons, the intestine, and the pharyngeal lumen, while expression profiling indicates that dgk-3 is expressed in the AFD thermosensory neurons as well as a small number of additional sensory neurons.
**WBGene00001650**	gon-1	F25H8.3	gon-1 encodes a functional metalloprotease that defines a new sub-family of secreted proteases known as MPT (metalloprotease with thrombospondin type 1 repeats); the other two members of this family are the

			bovine procollagen I N-protease (PINP) and the murine enzyme ADAMTS-1; gon-1 is essential for hermaphrodite gonadal morphogenesis and sequence homology with other metalloproteases suggests that it functions by remodeling the extracellular matrix; gon-1 is also required for protein transport from the endoplasmic reticulum (ER) to the Golgi, a function dependent on its C-terminal GON domain; GON-1 is expressed at high levels within the gonadal distal tip cell during migration and also in body wall muscle cells; in the distal tip cell, reporter fusion constructs expressing the GON domain localize to the endoplasmic reticulum, suggesting that GON-1 functions both intra- and extracellularly.
**WBGene00003002**	lin-13	C03B8.4	lin-13 encodes a large (2248-residue) nuclear protein with multiple zinc fingers of the C2H2 class and a LXCXE retinoblastoma protein-binding motif, that is required for survival through larval development and for negative regulation of vulval fates during postembryonic development.
**WBGene00003533**	nas-14	F09E8.6	nas-14 encodes an astacin-like metalloprotease; large-scale expression studies reveal that a nas-14::GFP promoter fusion is expressed in the pharynx.
**WBGene00003548**	nas-30	Y95B8A.1	nas-30 encodes an astacin family zinc metalloprotease with a single EGF domain and a CUB domain; nas-30 is expressed in the intestine and hypodermis.
**WBGene00003551**	nas-33	K04E7.3	nas-33 encodes an astacin family zinc metalloprotease; nas-33 is expressed in the intestine and hypodermis.
**WBGene00003602**	nhr-3	H01A20.1	nhr-3 encodes a member of the superfamily of nuclear receptors which is one of the most abundant class of transcriptional regulators; nuclear receptors have a well conserved DNA binding domain and a less conserved C-terminal ligand binding domain; nhr-3 has been identified and characterised as a gene affected by ethanol exposure in a microarray analysis of all *C. elegans* ORFs.
**WBGene00003628**	nhr-35	C07A12.3	
**WBGene00004319**	rbr-2	ZK593.4	rbr-2 encodes a histone H3 lysine 4 (H3K4) demethylase orthologous to budding yeast Jhd2p, Drosophila LID, and human JARID1A (OMIM:180202), JARID1B (OMIM:605393), JARID1C OMIM:314690, mutated in mental retardation), and JARID1D (OMIM:426000); RBR-2 regulates genome-wide levels of H3K4 trimethylation and is required in the germ line for normally short lifespan; recombinant His-RBR-2 specifically demethylates H3K4me3/me2 in vitro; rbr-2 is also required for reliable vulval development, and interacts genetically with CLK-2, GLP-1, LIN-15, and SEM-5; rbr-2(tm1231) homozygotes display excess trimethylated H3K4 (H3K4me3) and erratic vulval development (either vulvaless or multivulva); rbr-2(RNAi) animals show extended lifespans, a strong synthetic multivulva and H3K4me3 phenotype in a lin-15(n765ts) mutant background at permissive temperature, and abnormally slow growth with clk-2(mn159), glp-1(or178), or sem-5(n2019) mutant backgrounds; an RBR-2::GFP fusion protein is widely expressed and localizes to nuclei.

**WBGene00006648**	ttb-1	W03F9.5	
**WBGene00009164**	hrdl-1	F26E4.11	
**WBGene00010155**		F56F3.4	
**WBGene00010892**	dhhc-6	M18.8	
**WBGene00010923**	rle-1	M142.6	
**WBGene00010988**	metr-1	R03D7.1	R03D7.1 is orthologous to the human gene METHIONINE SYNTHASE (MTR; OMIM:156570), which when mutated leads to disease.
**WBGene00011004**		R04B5.6	R04B5.6 encodes one of two *C. elegans* sorbitol dehydrogense orthologs; by homology the product of R04B5.6 is predicted to catalyze the reversible oxidation of sorbitol to fructose in the presence of NAD+; in the embryo, an R04B5.6::gfp fusion is expressed in pharyngeal cells and head neurons.
**WBGene00012606**		Y38F1A.2	
**WBGene00012796**		Y43F4A.1	
**WBGene00014019**		ZK632.12	ZK632.12 encodes one of 12 *C. elegans* FYVE-domain containing proteins and is orthologous to mammalian Phafin2; as loss of ZK632.12 activity via RNAi screens results in no obvious defects, the precise role of ZK632.12 in *C. elegans* development and/or behavior is not yet known.
**WBGene00014075**	dhhc-4	ZK757.4	
**WBGene00016197**	pxl-1	C28H8.6	pxl-1 encodes proteins containing N-terminal LD motifs and C-terminal LIM domains that is the *C. elegans* ortholog of vertebrate paxillin; pxl-1 is required for organized myofilament structure in the pharynx and pharyngeal muscle contraction, and hence feeding and viability past the first larval stage; PXL-1 is expressed in body wall and pharyngeal muscle, as well as pharyngeal epithelial cells; PXL-1 localizes to sites of actin attachment, namely dense bodies, adhesion plaques, and M-lines in body wall muscle, and podosome-like structures in pharyngeal muscle; pxl-1 expression in pharyngeal muscle and marginal cells is sufficient to rescue the pharyngeal muscle defects and lethality of a pxl-1 mutant; PXL-1 physically interacts with muscle proteins DEB-1, UIG-1, LIM-8, UNC-96, UNC-95, and HUM-6.
**WBGene00017342**		F10D7.5	F10D7.5 encodes an ortholog of Drosophila NEURALIZED, and thus may participate in GLP-1/LIN-12 signalling; the promoter or 5' protein-coding regions of F10D7.5 are bound by DAF-16 in vivo, and F10D7.5 expression drops by roughly one-half in daf-16(mu86) mutants.
**WBGene00017644**	exc-9	F20D12.5	exc-9 encodes one of two *C. elegans* small LIM domain-containing proteins related to the mammalian CRIP

			(Cysteine-Rich Intestinal Protein) family of proteins; in *C. elegans*, exc-9 activity is required cell autonomously for proper organization of the apical cytoskeleton of the excretory canal cell, which is essential for maintaining the diameter of the excretory canal tubules; in addition, exc-9 is required for proper morphogenesis of the hermaphrodite and male tails; genetic analyses indicated that exc-9 likely functions upstream of exc-5, which encodes a guanine-nucleotide exchange factor, and downstream of exc-2 and sma-1, in regulating canal cell morphology; an EXC-9::GFP fusion protein is expressed in a number of cell types including the excretory canal cell, the tail spike, the uterine seam cell and anchor cell, the intestine, ALM and PLN neurons, and the nerve ring; in males, EXC-9::GFP expression is seen in a few tail neurons and weakly throughout the tail during larval development; EXC-9::GFP localizes to the cytoplasm; in yeast two-hybrid assays, EXC-9 interacts with CSN-5, a subunit of the COP9 signalosome likely involved in ubiquitin-mediated protein degradation.
**WBGene00017904**	lim-8	F28F5.3	lim-8 encodes proteins containing one PDZ and one LIM domain; in vitro binding and yeast two-hybrid assays indicate that LIM-8 can physically interact with myosin heavy chain A (MYO-3) as well as with UNC-96 and UNC-97, suggesting that LIM-8 is part of a structural component that links membrane attachment proteins to myosin thick filaments; lim-8(RNAi) in a hypersensitive rrf-3 background results in partially penetrant paralysis at mid-larval stages of development; a lim-8::gfp promoter fusion is expressed in pharyngeal and body wall muscles, as well as in vulva, spermathecae, anal sphincter and depressor muscles, head neurons, gonadal sheath, and the excretory canal; staining with LIM-8 antibodies reveals that in body wall muscle LIM-8 localizes, at least partially, to M-lines, around which myosin thick filaments are organized.
**WBGene00018966**		F56D2.5	
**WBGene00020496**	spat-3	T13H2.5	spat-3 encodes, by transcription from alternative promoters, two large (1092- and 2471-residues) proteins; while residues 162-228 of the larger SPAT-3 protein encode a Ring1 type of zinc-finger domain (like that seen in SEX COMBS EXTRA, an E3 ubiquitin-protein ligase RING1 protein of Drosophila melanogaster), the bulk of SPAT-3 has no obvious similarities to other proteins; consistent with its predicted role as a PRC1 component, spat-3 activity is required in vivo for histone H2A ubiquitination; spat-3 is also required for proper positioning of Ray 1 in the male tail, neuronal migration and axon extension, vulval development, and fully wild-type brood sizes; SPAT-3 is also specifically required for PAR protein-dependent cell-polarity, and this requirement is independent of PAR-2 activity; as with par-3, par-6, pkc-3, and cdc-42, inactivation of spat-3 (by RNAi or mutation) strongly suppresses the embryonic lethality of par-2(it5ts) at restrictive temperature; as with nos-3, spat-3 also suppresses par-2(lw32), a strong loss-of-function allele; while spat-3 par-2 double mutant embryos require PAR-1 for viability, PAR-1 does not regain posterior cortical localization in these embryos, but is instead diffused through their cytoplasm.
**WBGene00020694**	dhhc-11	T22E7.2	

**WBGene00021347**	rpb-10	Y37E3.3	Y37E3.3 encodes the *C. elegans* ortholog of the RNA polymerase II Rbp10 subunit; loss of Y37E3.3 activity via large-scale RNAi results in early embryonic lethality.
**WBGene00021924**		Y55F3AM.6	
**WBGene00022794**	snu-23	ZK686.4	
**WBGene00044071**	dhhc-14	D2021.2	

Mitochondrian associated genes

WormBase Gene ID	Gene Name	Sequence Name	Brief Description
**WBGene00000114**	alh-8	F13D12.4	alh-8 is orthologous to the human gene METHYLMALONATE-SEMIALDEHYDE DEHYDROGENASE (ALDH6A1; OMIM:603178), which when mutated leads to MMSDH deficiency.
**WBGene00000787**	cps-6	C41D11.8	cps-6 encodes an ortholog of human mitochondrial endonuclease G (EndoG) that promotes apoptosis, and is required to degrade nicked (TUNEL-positive) DNA in apoptotic cells; transgenic CPS-6 is localized to mitochondria, but transgenic CPS-6 lacking a mitochondrial localization sequence is found in nuclei, and nuclei may be the in vivo target of CPS-6 after its activation by CED-3; CPS-6 has magnesium-dependent nuclease activity in vitro and can degrade both single- and double-stranded DNA, as well as single-stranded RNA; CPS-6 also preferentially binds G-tract DNA in vitro; cps-6(sm116) and cps-6(RNAi) animals have delayed CED-3-induced apoptosis, and cps-6(sm116) suppresses a constitutively active ced-3 transgene; cps-6(sm116) can be transgenically rescued by mouse EndoG; CPS-6 binds WAH-1 (an apoptosis-inducing factor ortholog) in vitro, WAH-1 binding enhances CPS-6's endonuclease activity, and constitutive transgenic coexpression of cps-6 with wah-1 induces cell death not seen with constitutive expression of either cps-6 or wah-1 alone; CPS-6 binds CRN-1 (a flap endonuclease ortholog) in vitro, and may form a large complex in vivo with CRN-1, CRN-4, CRN-5, CYP-13, and WAH-1; CRN-1 enhances CPS-6's endonuclease activity in vitro, CPS-6 enhances CRN-1's gap-dependent endonuclease and 5'-3' exonuclease activities, and cps-6 is required for excess cell deaths induced by a crn-1 transgene.
**WBGene00001039**	dnj-21	T19B4.4	This gene encodes a protein containing a DnaJ ('J') domain.
**WBGene00001503**	fum-1	H14A12.2	fum-1 is orthologous to human FUMARASE (FH; OMIM:136850), which when mutated leads to fumarase deficiency; FUM-1A and FUM-1B are predicted to be mitochondrial.

**WBGene00007197**		B0513.5	
**WBGene00007330**		C05C10.3	C05C10.3 is orthologous to the human gene 3-OXOACID COA TRANSFERASE (also called succinyl-CoA:3-ketoacid CoA transferase; OXCT; OMIM:245050), which when mutated leads to episodic ketoacidosis.
**WBGene00008225**	nuaf-1	C50B8.3	
**WBGene00008414**		D2030.4	D2030.4 encodes the *C. elegans* ortholog of the NDUFB7/B18 subunit of the mitochondrial NADH dehydrogenase (ubiquinone) complex (complex I).
**WBGene00009559**	mtx-1	F39B2.11	
**WBGene00014202**	mmcm-1	ZK1058.1	mmcm-1 encodes an ortholog of human methylmalonyl-CoA mutase (MUT, mutated in methylmalonic acidemia; OMIM:609058); MMCM-1 enzyme, in vitro, kinetically resembles its human ortholog; mmcm-1 deletion mutants incorporate abnormally low levels of 1-[(14)C]-propionate into proteins; mmcm-1(RNAi) and mmcm-1 deletion mutant animals excrete abnormally high levels of methylmalonic acid into their culture medium when challenged with propionic acid; mmcm-1, in a lentiviral transgene, can partially rescue the mutant phenotype of human mut(o) fibroblasts; these data are consistent with the hypothesis that MMCM-1 participates in the conversion of propionyl-CoA to succinyl-CoA.
**WBGene00017261**	acl-6	F08F3.2	acl-6 encodes a mitochondrial glycerol-3-phosphate acyltransferase; acl-6 is predicted to play a role in triacylglycerol biosynthesis, and acl-6 expression is upregulated in animals during dauer larval development in response to direct exposure to daumone.
**WBGene00019900**	vdac-1	R05G6.7	

Transit peptide associated proteins

**WormBase Gene ID**	**Gene Name**	**Sequence Name**	**Brief Description**

**WBGene00000114**	alh-8	F13D12.4	alh-8 is orthologous to the human gene METHYLMALONATE-SEMIALDEHYDE DEHYDROGENASE (ALDH6A1; OMIM:603178), which when mutated leads to MMSDH deficiency.
**WBGene00000787**	cps-6	C41D11.8	cps-6 encodes an ortholog of human mitochondrial endonuclease G (EndoG) that promotes apoptosis, and is required to degrade nicked (TUNEL-positive) DNA in apoptotic cells; transgenic CPS-6 is localized to mitochondria, but transgenic CPS-6 lacking a mitochondrial localization sequence is found in nuclei, and nuclei may be the in vivo target of CPS-6 after its activation by CED-3; CPS-6 has magnesium-dependent nuclease activity in vitro and can degrade both single- and double-stranded DNA, as well as single-stranded RNA; CPS-6 also preferentially binds G-tract DNA in vitro; cps-6(sm116) and cps-6(RNAi) animals have delayed CED-3-induced apoptosis, and cps-6(sm116) suppresses a constitutively active ced-3 transgene; cps-6(sm116) can be transgenically rescued by mouse EndoG; CPS-6 binds WAH-1 (an apoptosis-inducing factor ortholog) in vitro, WAH-1 binding enhances CPS-6's endonuclease activity, and constitutive transgenic coexpression of cps-6 with wah-1 induces cell death not seen with constitutive expression of either cps-6 or wah-1 alone; CPS-6 binds CRN-1 (a flap endonuclease ortholog) in vitro, and may form a large complex in vivo with CRN-1, CRN-4, CRN-5, CYP-13, and WAH-1; CRN-1 enhances CPS-6's endonuclease activity in vitro, CPS-6 enhances CRN-1's gap-dependent endonuclease and 5'-3' exonuclease activities, and cps-6 is required for excess cell deaths induced by a crn-1 transgene.
**WBGene00001503**	fum-1	H14A12.2	fum-1 is orthologous to human FUMARASE (FH; OMIM:136850), which when mutated leads to fumarase deficiency; FUM-1A and FUM-1B are predicted to be mitochondrial.
**WBGene00007197**		B0513.5	
**WBGene00007330**		C05C10.3	C05C10.3 is orthologous to the human gene 3-OXOACID COA TRANSFERASE (also called succinyl-CoA:3-ketoacid CoA transferase; OXCT; OMIM:245050), which when mutated leads to episodic ketoacidosis.
**WBGene00008225**	nuaf-1	C50B8.3	
**WBGene00014202**	mmcm-1	ZK1058.1	mmcm-1 encodes an ortholog of human methylmalonyl-CoA mutase (MUT, mutated in methylmalonic acidemia; OMIM:609058); MMCM-1 enzyme, in vitro, kinetically resembles its human ortholog; mmcm-1 deletion mutants incorporate abnormally low levels of 1-[(14)C]-propionate into proteins; mmcm-1(RNAi) and mmcm-1 deletion mutant animals excrete abnormally high levels of methylmalonic acid into their culture medium when challenged with propionic acid; mmcm-1, in a lentiviral transgene, can partially rescue the mutant phenotype of human mut(o) fibroblasts; these data are consistent with the hypothesis that MMCM-1 participates in the conversion of propionyl-CoA to succinyl-CoA.
**WBGene00017261**	acl-6	F08F3.2	acl-6 encodes a mitochondrial glycerol-3-phosphate acyltransferase; acl-6 is predicted to play a role in triacylglycerol

			biosynthesis, and acl-6 expression is upregulated in animals during dauer larval development in response to direct exposure to daumone.

Glycoprotein associated genes

Gene > WormBase Gene ID	Gene > Gene Name	Gene > Sequence Name	Gene > Brief Description
**WBGene00000280**	cah-2	D1022.8	cah-2 encodes a predicted carbonic anhydrase.
**WBGene00001334**	ero-1	Y105E8B.8	ero-1 encodes an endoplasmic reticulum oxidoreductase that affects ER-stress response and affects reactive oxygen species levels.
**WBGene00001621**	glt-3	K08F4.4	glt-3 encodes an ortholog of glutamate/aspartate and neutral amino acid transporters.
**WBGene00001650**	gon-1	F25H8.3	gon-1 encodes a functional metalloprotease that defines a new sub-family of secreted proteases known as MPT (metalloprotease with thrombospondin type 1 repeats); the other two members of this family are the bovine procollagen I N-protease (PINP) and the murine enzyme ADAMTS-1; gon-1 is essential for hermaphrodite gonadal morphogenesis and sequence homology with other metalloproteases suggests that it functions by remodeling the extracellular matrix; gon-1 is also required for protein transport from the endoplasmic reticulum (ER) to the Golgi, a function dependent on its C-terminal GON domain; GON-1 is expressed at high levels within the gonadal distal tip cell during migration and also in body wall muscle cells; in the distal tip cell, reporter fusion constructs expressing the GON domain localize to the endoplasmic reticulum, suggesting that GON-1 functions both intra- and extracellularly.
**WBGene00002029**	hst-2	C34F6.4	hst-2 encodes the *C. elegans* ortholog of the heparan sulfate modifying enzyme 2O-sulfotransferase; by homology, HST-2 is predicted to function in heparan sulfate biosynthesis by catalyzing the chain-modifying sulfation of the C2 hydroxyl group of hexuronic acid; during development, hst-2 activity is required for normal body size, cell migration, and nervous system development; an hst-2::gfp reporter fusion is first expressed in embryos, continuing on through adulthood; expression is detected in many tissues, including the pharynx, hypodermis, muscles, vulva, and distal tip cells (DTCs) of the somatic gonad.
**WBGene00003533**	nas-14	F09E8.6	nas-14 encodes an astacin-like metalloprotease; large-scale expression studies reveal that a nas-14::GFP promoter fusion is expressed in the pharynx.

**WBGene00003548**	nas-30	Y95B8A.1	nas-30 encodes an astacin family zinc metalloprotease with a single EGF domain and a CUB domain; nas-30 is expressed in the intestine and hypodermis.
**WBGene00003551**	nas-33	K04E7.3	nas-33 encodes an astacin family zinc metalloprotease; nas-33 is expressed in the intestine and hypodermis.
**WBGene00004017**	phg-1	F27E5.4	
**WBGene00004048**	plx-2	K04B12.1	plx-2 encodes one of two *C. elegans* plexins, conserved transmembrane proteins that function as semaphorin receptors in both vertebrates and invertebrates; plx-2 activity is essential for proper male tail and epidermal morphogenesis and for normal axon guidance in a select group of neurons, including SDQL; genetic analyses indicate that plx-2 functions with mab-20/semaphorin-2a, efn-4/ephrin-4, and unc-129/TGF-beta to regulate male tail morphogenesis and with mab-20 and lad-2/L1CAM to mediate axon guidance; PLX-2 can bind MAB-20 and this interaction is enhanced in the presence of LAD-2; PLX-2::GFP reporters are expressed in a subset of neurons and epidermal cells, as well as a subset (1, 3, 5, and 7) of male sensory ray cell clusters.
**WBGene00007755**		C27A7.3	
**WBGene00008800**		F14E5.2	
**WBGene00009164**	hrdl-1	F26E4.11	
**WBGene00011482**	hpo-4	T05E11.6	
**WBGene00012796**		Y43F4A.1	
**WBGene00018418**		F44E2.4	
**WBGene00019682**		K12H4.7	
**WBGene00020509**	hex-1	T14F9.3	hex-1 encodes a beta-N-acetylhexosaminidase that is orthologous to the human gene CERVICAL CANCER PROTO-ONCOGENE 7 (HEXB; OMIM:606873), which when mutated leads to disease.
**WBGene00021852**		Y54F10AM.8	
**WBGene00022580**	iglr-2	ZC262.3	

Protease associated genes

Gene > WormBase Gene ID	Gene > Gene Name	Gene > Sequence Name	Gene > Brief Description
**WBGene00000083**	adt-2	F08C6.1	adt-2 encodes a member of the ADAMTS (a disintegrin and metalloprotease with thrombospodin motif) family of secreated metalloproteases; ADT-2 binds to extracellular matrix and is involved in remodeling of the extracellular matrix; homozygous adt-2 mutants die at the 3-fold stage of embryogenesis or during hatching; adult worms are about 20% reduced body length as compared to the wild type; adt-2 mutants have reduced lifespan with a median survival of 8 days from adulthood but have normal number of hypodermal nuclei; adt-2 is required for maintenance of body length as well as for increase in body length during growth stages; adt-2 regulate body size in part by modification of external cuticle; ADT-2 directly or indirectly regulates DBL-1 signaling activity; adt-2 expression is absent in embryos but is detected in glial cells associated with amphid, phasmid, labial and posterior deirids (PDE) sensory neurons during larval development and vulval tissue in L4 and adults.
**WBGene00000215**	asp-2	T18H9.2	asp-2 encodes aspartic protease.
**WBGene00001650**	gon-1	F25H8.3	gon-1 encodes a functional metalloprotease that defines a new sub-family of secreted proteases known as MPT (metalloprotease with thrombospondin type 1 repeats); the other two members of this family are the bovine procollagen I N-protease (PINP) and the murine enzyme ADAMTS-1; gon-1 is essential for hermaphrodite gonadal morphogenesis and sequence homology with other metalloproteases suggests that it functions by remodeling the extracellular matrix; gon-1 is also required for protein transport from the endoplasmic reticulum (ER) to the Golgi, a function dependent on its C-terminal GON domain; GON-1 is expressed at high levels within the gonadal distal tip cell during migration and also in body wall muscle cells;

			in the distal tip cell, reporter fusion constructs expressing the GON domain localize to the endoplasmic reticulum, suggesting that GON-1 functions both intra- and extracellularly.
**WBGene00003533**	nas-14	F09E8.6	nas-14 encodes an astacin-like metalloprotease; large-scale expression studies reveal that a nas-14::GFP promoter fusion is expressed in the pharynx.
**WBGene00003548**	nas-30	Y95B8A.1	nas-30 encodes an astacin family zinc metalloprotease with a single EGF domain and a CUB domain; nas-30 is expressed in the intestine and hypodermis.
**WBGene00003551**	nas-33	K04E7.3	nas-33 encodes an astacin family zinc metalloprotease; nas-33 is expressed in the intestine and hypodermis.
**WBGene00004401**	rom-2	C48B4.2	
**WBGene00011482**	hpo-4	T05E11.6	
**WBGene00012796**		Y43F4A.1	
**WBGene00019682**		K12H4.7	

Kinase associated genes

Gene > WormBase Gene ID	Gene > Gene Name	Gene > Sequence Name	Gene > Brief Description
**WBGene00000101**	aka-1	D1022.7	aka-1 encodes an A kinase anchor protein that has mutiple isoforms, all of which have a zinc-finger domain of the FYVE type; interacts with KIN-2 in vitro.
**WBGene00000102**	akt-1	C12D8.10	akt-1 encodes an ortholog of the serine/threonine kinase Akt/PKB; akt-1 genetically interacts with the insulin signaling pathway and functions to regulate such processes as dauer larval development and salt chemotaxis learning; AKT-1 binds calmodulin in vitro in a calcium-dependent manner; an AKT-1::GFP fusion protein is widely expressed beginning in late stage embryos and continuing through adulthood; expression is seen in head, tail, and dorsal and ventral cord neurons, with additional expression seen in other cells including those of the pharynx, hypodermis, intestine, and spermatheca; two alleles of akt-1 (sa573 and sa700) have a Daf-c mutant phenotype at 27 degrees C (Hid phenotype).
**WBGene00000960**	dgk-3	F54G8.2	dgk-3 encodes a diacylglycerol kinase that is the *C. elegans* ortholog of mammalian DGK-beta; dgk-3 activity is required for regulation of long-term thermotactic behavioral plasticity and for regulation of olfactory adaptation; large-scale expression studies have reported dgk-3 expression in head neurons, the intestine, and the pharyngeal lumen, while expression profiling indicates that dgk-3 is expressed in the AFD thermosensory

			neurons as well as a small number of additional sensory neurons.
**WBGene00002188**	kgb-2	ZC416.4	
**WBGene00003186**	mek-2	Y54E10BL.6	mek-2 encodes a MAP kinase kinase; MEK-2 functions as a core component of Ras-mediated signal transduction pathways that regulate several biological processes including larval development, vulval development, cell migration, meiotic cell cycle progression, olfaction, and pathogen defense.
**WBGene00003247**	mig-15	ZC504.4	The mig-15 gene encodes a Nck-interacting kinase (NIK) that is required to inhibit premature branching of commissures.
**WBGene00003961**	pct-1	C07G1.3	
**WBGene00004044**	plk-3	F55G1.8	plk-3 encodes one of three *C. elegans* polo-like kinases; microarray analyses indicate that plk-3 transcripts are expressed maternally and downregulated in gonadal tissue mutant for deps-1, which encodes a novel, P granule-associated protein that promotes P granule assembly and germline RNA interference.
**WBGene00004894**	sms-3	Y22D7AL.8	
**WBGene00005078**	src-2	F49B2.5	src-2 encodes a non-receptor protein tyrosine kinase that, along with src-1, is one of two *C. elegans* Src family kinase members; although src-2(RNAi) alone results in no overt phenotype, src-2(RNAi) performed in the background of retinoblastoma pathway mutants results in embryonic and larval lethality, as well as sterility; a src-2::GFP reporter fusion is expressed in pharyngeal muscles, the vulva, and some cells around anus; overexpression of SRC-2 in yeast cell results in an overall increase in protein tyrosine phosphorylation levels, which is suppressed by co-expression with CSK-1, a predicted negative regulator of SRC-2 activity.
**WBGene00006461**	tag-96	M01D7.4	tag-96 encodes a galactokinase that is a member of the GHMP family of kinases and closely related to the vertebrate galactokinase 2 (GALK2) carbohydrate kinases.
**WBGene00006786**	unc-51	Y60A3A.1	unc-51 encodes a serine/threonine protein kinase orthologous to *Saccharomyces cerevisiae* autophagy protein Atg1p and the vertebrate ULK proteins; unc-51 is required for axon outgrowth along the anterior-posterior axis and sex myoblast migration; in regulating axon outgrowth, UNC-51 functions together with the VAB-8 kinesin-like protein and UNC-14, both of which physically interact with, and are phosphorylated by, UNC-51, and with the UNC-5 Netrin receptor, whose subcellular localization in neurons is regulated by UNC-51 and UNC-14; in addition, UNC-51 is required for normal dauer morphogenesis of daf-2 mutant animals; UNC-51 is expressed in all *C. elegans* neurons and in body wall and pharyngeal muscles; in neurons, an UNC-51::GFP fusion protein shows punctate cytoplasmic localization in axons and cell bodies and partial co-localization with UNC-14 and UNC-5.
**WBGene00007745**		C26D10.4	
**WBGene00008311**	rskn-2	C54G4.1	

**WBGene00008746**		F13E6.2	
**WBGene00019362**	cdk-2	K03E5.3	cdk-2 encodes the *C. elegans* ortholog of cyclin-dependent kinase 2; along with CYE-1 (cyclin E), CDK-2 is required for the G1/S transition in somatic cells, centrosome assembly and polarity establishment in the embryo, proliferation of germ cells, and suppression of terminal differentiation in quiescent cells after asymmetric division; CYE-1 and CDK-2 also regulate the germline mitosis/meiosis decision through post-translational regulation of GLD-1, likely direct phosphorylation.

Serine/threonine kinase associated genes

Gene > WormBase Gene ID	Gene > Gene Name	Gene > Sequence Name	Gene > Brief Description
**WBGene00000102**	akt-1	C12D8.10	akt-1 encodes an ortholog of the serine/threonine kinase Akt/PKB; akt-1 genetically interacts with the insulin signaling pathway and functions to regulate such processes as dauer larval development and salt chemotaxis learning; AKT-1 binds calmodulin in vitro in a calcium-dependent manner; an AKT-1::GFP fusion protein is widely expressed beginning in late stage embryos and continuing through adulthood; expression is seen in head, tail, and dorsal and ventral cord neurons, with additional expression seen in other cells including those of the pharynx, hypodermis, intestine, and spermatheca; two alleles of akt-1 (sa573 and sa700) have a Daf-c mutant phenotype at 27 degrees C (Hid phenotype).
**WBGene00002188**	kgb-2	ZC416.4	
**WBGene00003186**	mek-2	Y54E10BL.6	mek-2 encodes a MAP kinase kinase; MEK-2 functions as a core component of Ras-mediated signal transduction pathways that regulate several biological processes including larval development, vulval development, cell migration, meiotic cell cycle progression, olfaction, and pathogen defense.
**WBGene00003247**	mig-15	ZC504.4	The mig-15 gene encodes a Nck-interacting kinase (NIK) that is required to inhibit premature branching of commissures.
**WBGene00003961**	pct-1	C07G1.3	
**WBGene00004044**	plk-3	F55G1.8	plk-3 encodes one of three *C. elegans* polo-like kinases; microarray analyses indicate that plk-3 transcripts are expressed maternally and downregulated in gonadal tissue mutant for deps-1, which encodes a novel, P granule-associated protein that promotes P granule assembly and germline RNA interference.
**WBGene00006786**	unc-51	Y60A3A.1	unc-51 encodes a serine/threonine protein kinase orthologous to *Saccharomyces cerevisiae* autophagy protein Atg1p and the vertebrate ULK proteins; unc-51 is required for axon outgrowth along the anterior-posterior

			axis and sex myoblast migration; in regulating axon outgrowth, UNC-51 functions together with the VAB-8 kinesin-like protein and UNC-14, both of which physically interact with, and are phosphorylated by, UNC-51, and with the UNC-5 Netrin receptor, whose subcellular localization in neurons is regulated by UNC-51 and UNC-14; in addition, UNC-51 is required for normal dauer morphogenesis of daf-2 mutant animals; UNC-51 is expressed in all *C. elegans* neurons and in body wall and pharyngeal muscles; in neurons, an UNC-51::GFP fusion protein shows punctate cytoplasmic localization in axons and cell bodies and partial co-localization with UNC-14 and UNC-5.
**WBGene00008311**	rskn-2	C54G4.1	
**WBGene00016421**	cdc-7	C34G6.5	
**WBGene00019362**	cdk-2	K03E5.3	cdk-2 encodes the *C. elegans* ortholog of cyclin-dependent kinase 2; along with CYE-1 (cyclin E), CDK-2 is required for the G1/S transition in somatic cells, centrosome assembly and polarity establishment in the embryo, proliferation of germ cells, and suppression of terminal differentiation in quiescent cells after asymmetric division; CYE-1 and CDK-2 also regulate the germline mitosis/meiosis decision through post-translational regulation of GLD-1, likely direct phosphorylation.
**WBGene00022616**	hsd-3	ZC449.6	

Transport associated genes

WormBase Gene ID	Gene Name	Sequence Name	Brief Description
**WBGene00000054**	acr-15	F25G6.4	A homolog of an alpha type nicotinic acetylcholine receptor subunit involved in the mediation of fast synaptic transmission at neuromuscular junctions.
**WBGene00000367**	cca-1	C54D2.5	cca-1 encodes a calcium channel alpha subunit that is homologous to vertebrate T-type calcium channel alpha 1 subunits; CCA-1 is required for regulation of pharyngeal pumping, specifically for the efficient initiation of action potentials in the pharynx in response to excitatory inputs; a CCA-1::GFP fusion protein is expressed strongly in pharyngeal muscle as well as in many neurons, including particular subsets of neurons in the head, pharynx, ventral nerve cord, and anal ganglia.
**WBGene00000464**	ceh-44	Y54F10AM.4	ceh-44 encodes at least three proteins via alternative splicing; the CEH-44A protein is an ortholog of the CUX class of CUT homeodomain proteins, expressed in early embryos (mostly anterior in the comma stage), in the larval nerve ring and the adult gonad; the CUX class includes Drosophila CUT, human CUTL1 and

			human CUTL2; ceh-44 is a complex gene, in which two alternatively spliced protein products completely lack the homeodomain and cut domain, and instead form a different protein, CASP; in vertebrates, this alternative splicing also occurs, while in Drosophila Casp as been lost; CASP proteins are also found in plants and fungi (though not associated with homeodomains); like its orthologs, CEH-44A has three cut domains upstream of the homeodomain; the cut domain is a DNA binding domain.
**WBGene00001039**	dnj-21	T19B4.4	This gene encodes a protein containing a DnaJ ('J') domain.
**WBGene00001334**	ero-1	Y105E8B.8	ero-1 encodes an endoplasmic reticulum oxidoreductase that affects ER-stress response and affects reactive oxygen species levels.
**WBGene00001365**	exc-4	Y105E8A.22	exc-4 encodes a highly conserved member of the chloride intracellular channel (CLIC) family of anion channels; EXC-4 is required during early stages of excretory cell tubulogenesis and appears also to play a role in vulval and seam cell development, which subsequently affects viability, fertility, and locomotion; EXC-4 is localized to the apical/lumenal membrane of the excretory cell and is also expressed in the hypodermis, vulva, and rectal gland cell.
**WBGene00001621**	glt-3	K08F4.4	glt-3 encodes an ortholog of glutamate/aspartate and neutral amino acid transporters.
**WBGene00002151**	irk-3	K04G11.5	
**WBGene00002173**	itr-1	F33D4.2	itr-1 encodes a putative inositol (1,4,5) trisphosphate receptor that affects the defecation cycle and pharyngeal pumping, and also affects ovulation in a pathway downstream of LET-23; interacts with UNC-54 in vivo, and is expressed in the adult intestine, pharynx, excretory cell, germ line, and spermatheca, with limited neuronal expression.
**WBGene00002260**	lbp-8	T22G5.6	lpb-8 belongs to a family of small intracellular fatty acid binding proteins (FABPs) that includes As-p18, found in the parasitic nematode *Ascaris suum*; lpb-8 shares 55% identity with bovine heart FABP but only 25% identity with As-p18 and lacks a secretory signal
**WBGene00003774**	nmr-1	F07F6.6	nmr-1 encodes an NMDA-type ionotropic glutamate receptor subunit that affects the duration of forward movement which is important during foraging behavior, and also affects osmotic avoidance; the slow kinetics typical of NMDA-dependent currents are likely important for its effect on forward movement.
**WBGene00003905**	pad-1	Y18D10A.13	The pad-1 gene encodes a highly conserved, but unfamiliar, protein that is required for embryonic development.
**WBGene00004793**	shw-3	R186.5	
**WBGene00006798**	unc-64	F56A8.7	unc-64 encodes syntaxin, a plasma membrane receptor for intracellular vesicles that is orthologous to vertebrate syntaxin 1A (OMIM:186590) and Drosophila Syx1A; UNC-64 is required for normal locomotion and possibly also for insulin secretion; as an essential component of the core synaptic vesicle fusion machinery, UNC-64 interacts with UNC-13, a diacylglycerol-binding protein, and SNB-1/synaptobrevin; UNC-64 trafficking from the endoplasmic reticulum to the plasma membrane is mediated by UNC-18, an SM

			(Sec1, Munc18) family member; unc-64 mutations can be suppressed by mutations in slo-1, a calcium-activated potassium channel; UNC-64 is expressed ubiquitously in the nervous system and in secretory cells such as the vulval uv1 cell and the excretory gland cells.
**WBGene00008414**		D2030.4	D2030.4 encodes the *C. elegans* ortholog of the NDUFB7/B18 subunit of the mitochondrial NADH dehydrogenase (ubiquinone) complex (complex I).
**WBGene00008570**	kcnl-2	F08A10.1	
**WBGene00009559**	mtx-1	F39B2.11	
**WBGene00009882**	vha-17	F49C12.13	vha-17 (aka 'fus-1') encodes an an ortholog of subunit e of the membrane-bound (V0) domain of vacuolar proton-translocating ATPase (V-ATPase); VHA-17, like VHA-1 and VHA-12, antagonizes EFF-1-mediated cell fusion in hypodermal cells; VHA-17 is expressed in gut cells of comma stage embryos, and in excretory cell and apical membranes of gut cells at later embryonic stages; VHA-17 is found in gut granules (a type of lysosome, expected to require acidification), and glo-1 mutant intestines fail to show punctate VHA-17; VHA-17 is required for viability, but this is at least somewhat genetically separable from VHA-17's antifusogenic activity.
**WBGene00013672**	catp-1	Y105E8A.12	catp-1 encodes an alpha subunit of the Na+/K+- and H+/K+-pump P-type ATPase family; catp-1 was identified in screens for animals resistant to developmental defects seen in larvae treated with the nicotinic agonist DMPP; genetic analyses indicate that catp-1 likely functions together with the let-60/Ras and daf-2/insulin-like receptor signaling pathways to regulate L2 larval developmental timing and dauer formation in a manner independent of its ATPase domain and thus, predicted transporter activity; a catp-1::gfp promoter fusion is expressed in hypodermal cells and in the excretory duct cell; hypodermal-specific catp-1(RNAi) confers partial resistance to DMPP, suggesting that hypodermal CATP-1 expression mediates DMPP toxicity.
**WBGene00014030**	glb-1	ZK637.13	glb-1 encodes a globin; glb-1 transcription is uninduced by anoxia, but repressed by DAF-2 signalling in a DAF-16 dependent manner; glb-1 is expressed in distal germline, late embryonic cells that are probably hypodermal precursors, and young adults; glb-1 transcription is lower in L3 larvae than in adults, while in adults it is higher than that of most other globins; glb-1 has no obvious function in mass RNAi assays.
**WBGene00015165**		B0361.11	
**WBGene00015969**	glb-6	C18C4.9	glb-6 encodes a novel globin-like protein; GLB-6 is expressed in neurons and glb-6 expression is downregulated in a daf-2(e1370) mutant background under normoxic conditions; biochemical characterization of GLB-6 indicates that CO, NO, and CN- do not bind to the heme in GLB-6 and that GLB-6 demonstrates a low redox potential and rapid, two-state autoxidation kinetics.
**WBGene00016184**	glb-9	C28F5.2	glb-9 encodes a globin with no obvious function in mass RNAi assays.
**WBGene00019481**	cogc-6	K07C11.9	cogc-6 encodes an ortholog of mammalian COG-6, a subunit of lobe B of the conserved oligomeric Golgi complex (COGC); COGC-6 is weakly required for normal gonadal distal tip cell migration, a process that

			also requires seven other orthologs of COGC subunits; like other lobe B subunits in both *C. elegans* and *S. cerevisiae*, COGC-6 is only partially required for normal function, while lobe A subunits are strongly required in either worms or yeast.
**WBGene00019900**	vdac-1	R05G6.7	
**WBGene00022295**	cng-2	Y76B12C.1	
